# Fingerprinting Mediterranean hurricanes using pre-event thermal drops in seawater temperature

**DOI:** 10.1038/s41598-024-58335-w

**Published:** 2024-04-05

**Authors:** Giovanni Scardino, Mario Marcello Miglietta, Alok Kushabaha, Elisa Casella, Alessio Rovere, Giovanni Besio, Alfio Marco Borzì, Andrea Cannata, Gianfranco Mazza, Gaetano Sabato, Giovanni Scicchitano

**Affiliations:** 1 Department of Earth and Geoenvironmental Sciences, University of Bari Aldo Moro, 70125 Bari, Italy; 2 Interdepartmental Research Center for Coastal Dynamics, University of Bari Aldo Moro, 70125 Bari, Italy; 3 Institute of Atmospheric Sciences and Climate (CNR-ISAC), National Research Council of Italy, Padua, Italy; 4 IUSS - School for Advanced Studies, Pavia, Italy; 5 Department of Environmental Sciences, Informatics and Statistics, Ca’ Foscari University of Venice, Venezia, Italy; 6 Department of Civil, Chemical and Environmental Engineering, University of Genoa, Genoa, Italy; 7 Department of Biological, Geological and Environmental Sciences, University of Catania, Catania, Italy; 8 Osservatorio Etneo, Istituto Nazionale di Geofisica e Vulcanologia - Sezione di Catania, Catania, Italy; 9 Area Marina Protetta del Plemmirio, 96100 Siracusa, Italy; 10 MARUM - Center for Marine Environmental Sciences, University of Bremen, Bremen, Germany

**Keywords:** Climate sciences, Environmental sciences, Natural hazards

## Abstract

Extreme atmospheric-marine events, known as medicanes (short for “Mediterranean hurricanes”), have affected the Mediterranean basin in recent years, resulting in extensive coastal flooding and storm surges, and have occasionally been responsible for several casualties. Considering that the development mechanism of these events is similar to tropical cyclones, it is plausible that these phenomena are strongly affected by sea surface temperatures (SSTs) during their development period (winter and autumn seasons). In this study, we compared satellite data and the numerical reanalysis of SSTs from 1969 to 2023 with in situ data from dataloggers installed at different depths off the coast of southeastern Sicily as well as from data available on Argo floats on the Mediterranean basin. A spectral analysis was performed using a continuous wavelet transform (CWT) for each SST time series to highlight the changes in SSTs prior to the occurrence of Mediterranean Hurricanes as well as the energy content of the various frequencies of the SST signal. The results revealed that decreases in SST occurred prior to the formation of each Mediterranean hurricane, and that this thermal drop phenomenon was not observed in intense extra-tropical systems. The spectral analyses revealed that high CWT coefficients representing high SST energy contents were observed before the occurrence of a Mediterranean hurricane. This information may provide a useful fingerprint for distinguishing Mediterranean hurricanes from common seasonal storms at the onset of these events.

## Introduction

The Mediterranean region is the epicenter for many extreme atmospheric-marine events,whose intensification or increase in recurrence interval is often associated with climate change^1^. For example, Mediterranean cyclones^2^ are typically induced by the southward deviations of the polar jet, leading to baroclinic instability; this developmental mechanism, typical of extratropical cyclones, can intensify pre-existent surface cyclonic circulation^3,4^ and enhance convection near the center of the cyclone. The latent heat released during convection can further depress the central sea-level pressure (SLP) and intensify cyclonic circulation through diabatically produced potential vorticity (PV) anomalies in the lower-mid troposphere^5,6^.

Different patterns of evolution can be observed as a cyclone reaches a maturity stage^7,8^. In some cases, the air-sea interaction and the heat released during convection are the main mechanisms by which cyclones intensify as they mature: these cyclones, known as Mediterranean hurricanes (and commonly called ‘medicanes’), which are among the most intense Mediterranean cyclones^9,10^. Model simulations have been used in an effort to reproduce the effects of Mediterranean hurricanes, using the cyclone phase-space analysis to determine the evolution of their thermodynamic characteristics under changing climate conditions^11,12^. According to Pytharoulis^13^ and Stathopolous et al.^14^, sea surface temperature (SST) values, which are strongly affected by global warming, appear to be an important factor that influences the intensification of Mediterranean hurricanes. Cavicchia et al.^15^ identified the expected values of several environmental parameters that signaled the occurrence of Mediterranean hurricanes. More recently, Gutierrez-Fernandez et al.^16^ identified the typical values of some large-scale and mesoscale environmental parameters to characterize the environments in which Mediterranean hurricanes and other types of warm-core cyclones develop. In this study, we analyzed SSTs and satellite observations associated with the occurrence of cyclones. Significant changes were observed in the SST time series values during cyclone occurrence, with high SST values observed during autumn as well as a thermal drop in excess of 1.6 °C prior to the occurrence of Mediterranean hurricanes. SST satellite observations and numerical reanalysis were compared with data from Argo floats as well as from a thermal sensor installed on the coast of Siracusa (Sicily, southern Italy), which showed SST decreased during Medicanes Ianos (2020) and Apollo (2021), which had cyclone tracks that bordered the Sicilian coast. The results of this study suggest that changes in SST can be an important indicator for the early warning of potential Mediterranean hurricanes.

### Cyclone dynamics and Mediterranean hurricanes

**Figure 1:**
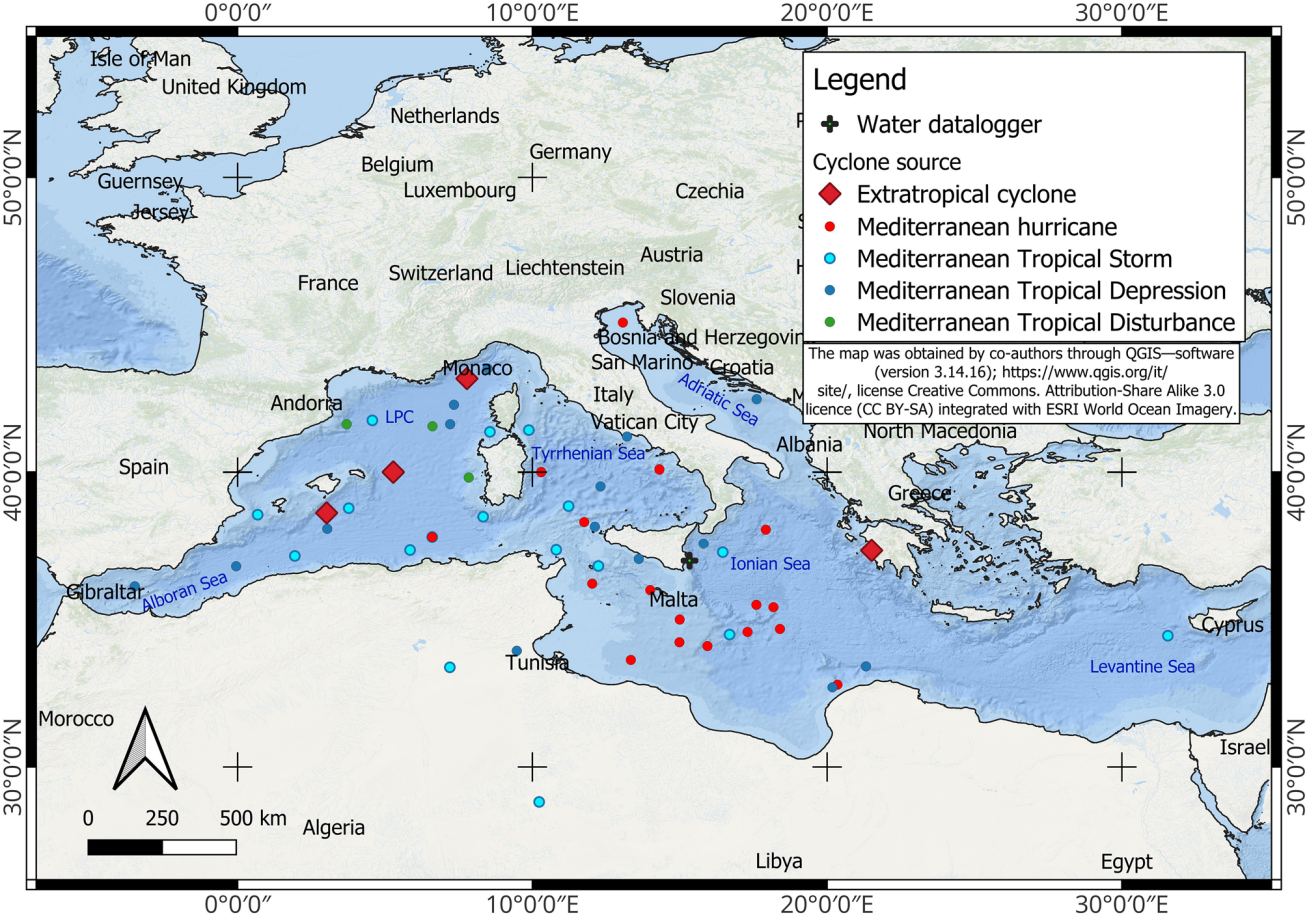
Locations of cyclone formation in the Mediterranean basin. The cyclone events presented here are reported in Table S1 of Supplementary Material. Blue labels indicate the main sub-basins. *LPC* Liguro–Provencal and Catalan sub-basins (modified by Shaltout and Omstedt^36^).

The occurrence of cyclone events in the Mediterranean basin is strongly related to its complex topographical features as well as the unique air-sea interactions in the region^17,18^. Mediterranean cyclones may form and develop in different ways; a comprehensive review is provided by Flaounas et al.^2^. These cyclones, which have marked seasonal cycles^19^, are more frequent in autumn and winter and generally affect the northern and central areas of the Mediterranean basin. The most intense cyclones occur in the northwest Mediterranean as well as in the Ionian, Aegean, and Black seas^2^ (Fig. 1). Two main categories of cyclones occur in the Mediterranean basin: tropical-like cyclones and extratropical cyclones. Tropical-like cyclones are symmetric, deep, warm-core cyclones sustained by air-sea interaction processes and latent heat released by the sea; while extratropical cyclones are asymmetric, cold-core cyclones sustained by baroclinic instability.

Due to the limited extent of the basin, Mediterranean cyclones have lower intensities, smaller sizes, and shorter durations than their mid-latitude counterparts^2,20^, and the early stages of these cyclones are often characterized by the interaction of upper-tropospheric PV streamers with low-level baroclinic areas^21,22^; such conditions are favorable to the development of baroclinic instability, possibly modulated by land-sea differences^17^. Mediterranean orography also plays a role; in particular, the reliefs of northwest Africa (i.e. the Atlas mountains) and the chains on the northern edge of the Mediterranean basin are responsible for lee cyclogenesis in a two-step process^23,24^. In the first phase, cold air advection is retarded on the upwind slopes, while positive thermal anomalies and low pressure are produced on the lee side^2^. In the second phase, the reliefs affect the baroclinic instability of the area, while heat fluxes, latent heat release, and low-level PV filaments influence the formation of the low-pressure area on the lee side^25,26^.

Some Mediterranean cyclones exhibit visual and physical similarities with tropical cyclones^27^. Tous and Romero^28^ suggested that Mediterranean hurricanes can be distinguished from other Mediterranean cyclones by their visual resemblance to tropical cyclones in satellite imagery: in particular, Mediterranean hurricanes can be recognized by their symmetric structure and the presence of a cloud-free area with a central ‘eye’ surrounded by bands of spiral clouds^29^. Emanuel (2005) showed that the intensification of a winter cyclone in the Ionian Sea in 1995 could be explained in terms of wind-induced surface heat exchange (WISHE), a developmental mechanism similar to tropical cyclones; consequently, he christened this category of cyclones “medicanes”, short for Mediterranean hurricane. However, Fita and Flaounas^30^ studied Medicane Zeo (December 2005) and suggested that some Mediterranean hurricanes are hybrid systems and can exhibit features of both tropical and extra-tropical cyclones; consequently, they believed that Mediterranean hurricanes should be classified as part of the wider category of subtropical cyclones^2^. In general, Mediterranean hurricanes exhibit features similar to those of tropical systems for a short period near the end of their lifetime, while exhibiting extra-tropical features in their initial stages^27,31^. They generally have a horizontal extension of a few hundred kilometers and an intensity comparable to Category 1 on the Saffir-Simpson hurricane wind scale (Category 2, in the case of Ianos)^32^. Mediterranean hurricanes usually occur once or twice a year between September and February^23,33,34^.

Mediterranean tropical-like cyclones can be classified on the Saffir-Simpson scale for tropical cyclones based on their wind speed and mean sea-level pressure (MSLP)^27,35^ (Table 1).

**Table 1:** Classification of tropical-like cyclones based on wind speed and mean sea-level pressure (MSLP) for the identification of the strongest events in the Mediterranean basin.

	Tropical-like cyclones			
	Mediterranean tropical disturbance	Mediterranean tropical depression	Mediterranean tropical storm	Mediterranean hurricane
Wind speed	Less than 45 km/h	45–61 km/h	63–99 km/h	100–152 km/h
MSLP	–	1006–1015 hPa	994–1005 hPa	974–993 hPa

Exceptionally strong cyclones have also been recorded in association with extra-tropical cyclones, such as storm Vaia, which occurred between 27 and 30 October 2018 and exhibited wind speeds and air pressures comparable to Mediterranean hurricanes. Although they represent the large majority of Mediterranean cyclones, this study only considered a few strong extra-tropical cyclones when comparing these storm events to Mediterranean hurricanes.

An important parameter that influences the stability of cyclones is the air-sea temperature gradient^15^. SST is suggested to be an important factor in the mature stages of cyclone development^29^. According to Pytharoulis^13^ and Miglietta et al.^31^ high SSTs promote stronger sea surface fluxes and, consequently, stronger latent heat release due to convection needed for Mediterranean hurricane intensification. In addition, the intrusion of cold air can increase instability, making the development of Mediterranean hurricanes possible even in the presence of rather low SSTs. The trend of Mediterranean SSTs varies considerably with season, ranging between 9.7–17.7 °C in winter, 15.8–22.1 °C in spring, 20.8–28.3 °C in summer, and 15.1–23.4 °C in autumn^36^. In addition, SST varies spatially over the Mediterranean basin, with SSTs of less than 17.1 °C frequently occurring in the Gulf of Lion and the northern Adriatic sub-basin, in contrast to the east of the Levantine sub-basin, which commonly exhibits temperatures in excess of 22.4 °C. Such temperatures are appropriate for the development of Mediterranean hurricanes, which form within a temperature range between 15 and 27 °C; this is in contrast to tropical cyclones, which generally occur at SSTs above a threshold of 26.5 °C^29^.

Mediterranean hurricanes are characterized by a marked seasonality. Despite the higher SSTs compared to the rest of the year, Mediterranean hurricanes do not develop in the summer months; instead, they most often occur in autumn, when SSTs are slightly lower than summer values and the mixed layer depth (MLD) deepens, or in winter, when SSTs are low and the MLD has reached its maximum^31,37^.

## Results

**Figure 2:**
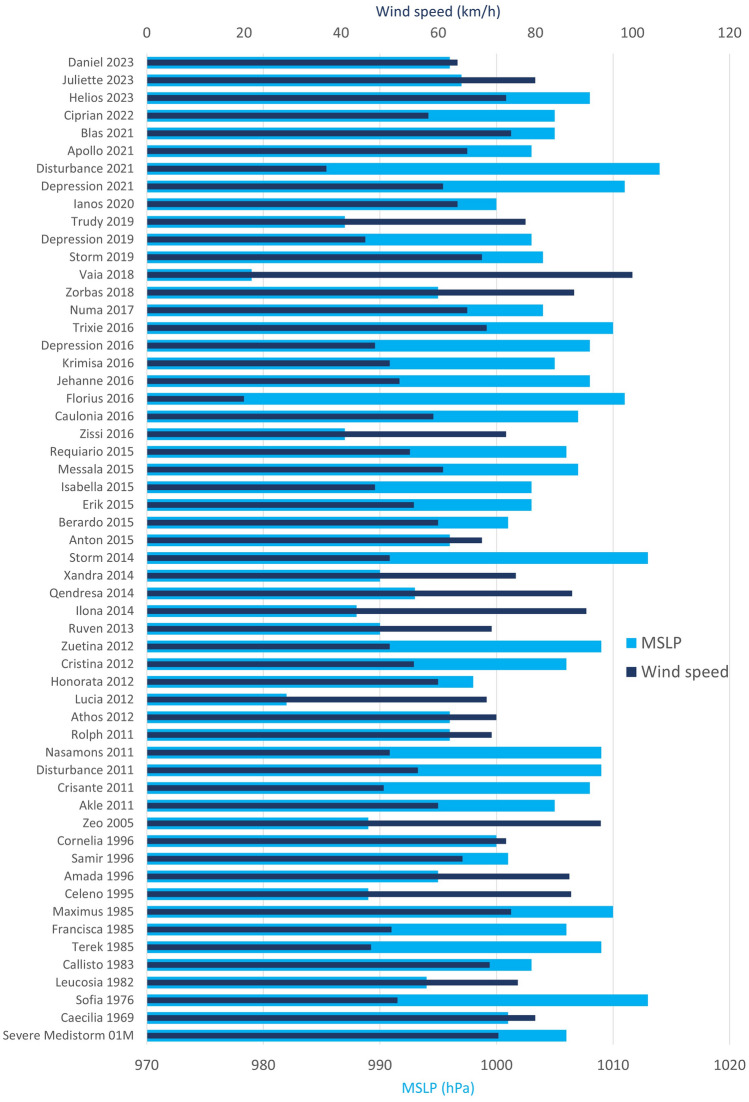
Wind speed and MSLP extracted from ERA5 hourly dataset for selected Mediterranean cyclones.

Mediterranean cyclones with tropical characteristics were classified using the intensity scale usually adopted for tropical cyclones. MSLP was extracted from ERA5 reanalysis data for each event listed in Table S1, with the wind speed evaluated from the u-v components of 10 m winds. The results revealed increased wind speeds during the occurrence of Mediterranean hurricanes, comparable with Category 1 events on the Saffir–Simpson hurricane wind scale (Fig. 2) and the intense extra-tropical cyclones selected.

Thermal changes were calculated from different datasets using the difference in SST in the 1 × 1° cyclogenesis area during and 10 days before cyclone occurrence (see Material and methods).

**Figure 3:**
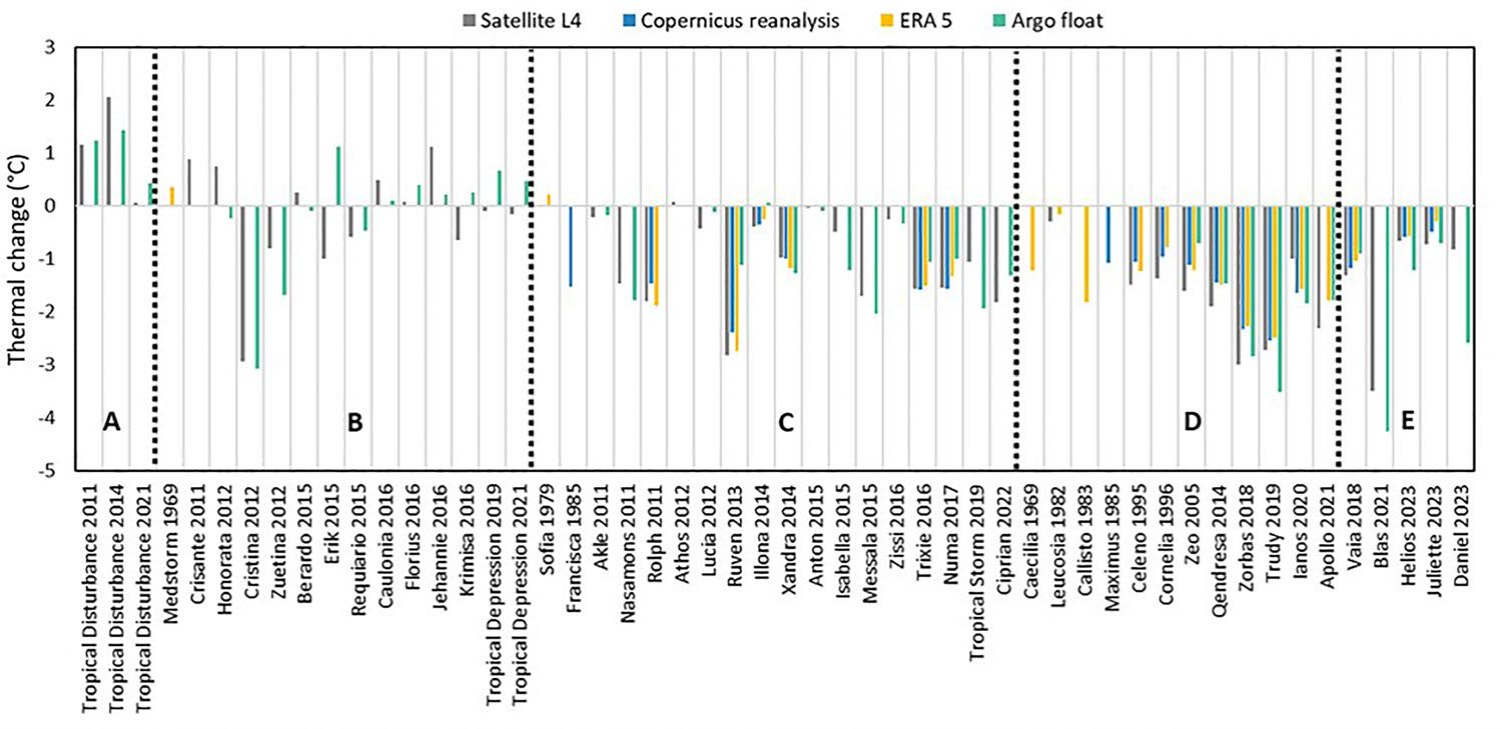
Thermal changes observed for selected Mediterranean cyclone events. Temperatures were compared between the last day of the cyclone’s lifetime and 10 days before cyclone occurrence at the location of cyclogenesis location through a variety of different datasets (Satellite L4, Copernicus reanalysis, ERA5, and Argo floats). A full list of events is presented in Table S1 and is categorized as follows—(**A**): Mediterranean tropical disturbance; (**B**): Mediterranean tropical depression; (**C**): Mediterranean tropical storm; (**D**): Mediterranean hurricane (i.e., medicane); (**E**): extratropical cyclone.

An analysis of the thermal changes observed for the events reported in Table S1 revealed that the most intense cyclones were characterized by a thermal drop equal to or greater than 1.6 °C. The weather systems associated with the greatest thermal drops were the Mediterranean hurricanes, with thermal drops in a range of 2–3 °C. Conversely, satellite observations and ERA5 reanalysis consistently showed that the magnitude of thermal drops was greater for Mediterranean hurricanes compared to extra-tropical cyclones and Mediterranean tropical storms (Fig. 3).

**Figure 4:**
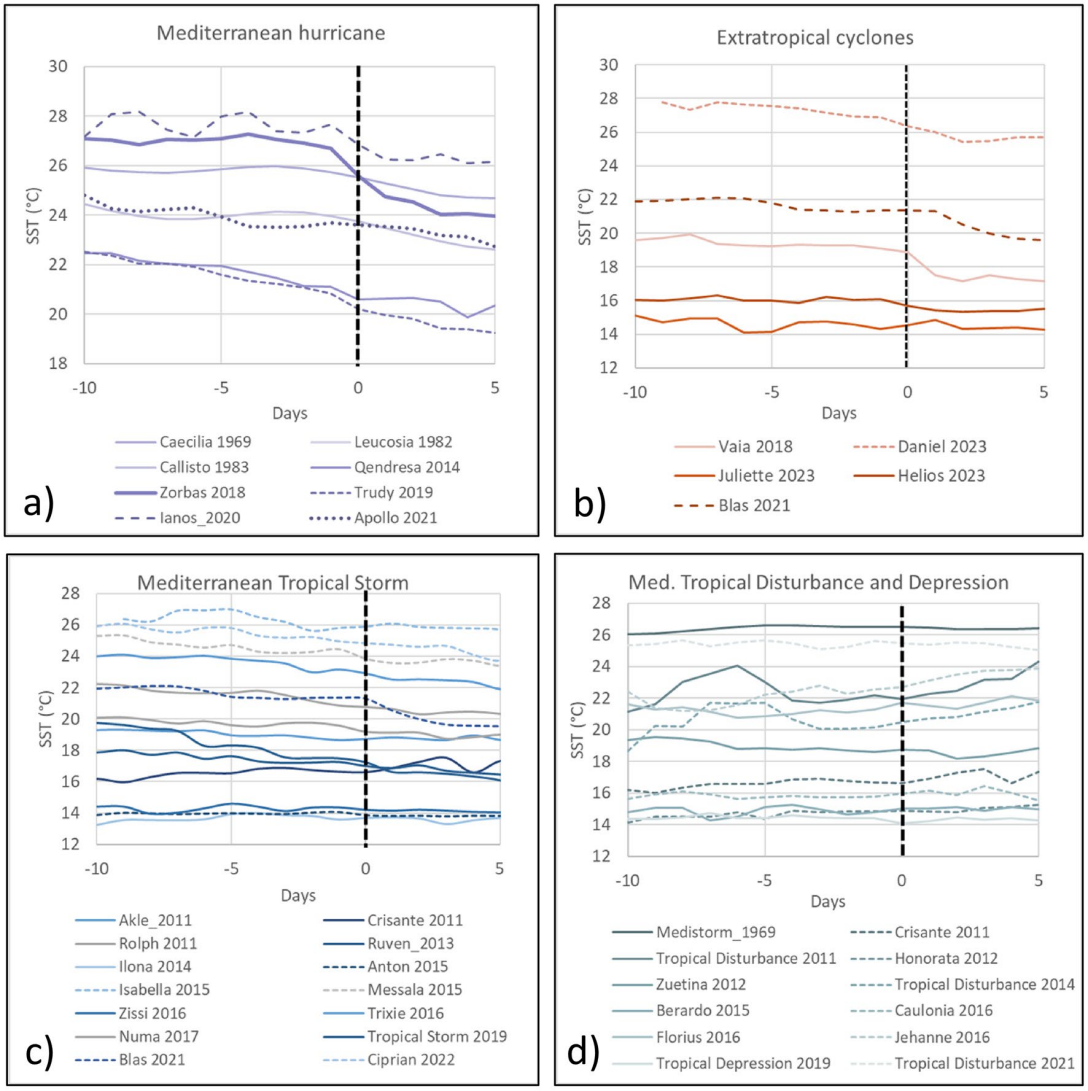
SST time series for cyclone events listed in Table S1. The origin of the x-axis corresponds to the beginning of the event. (**a**) SST time-series data for Mediterranean hurricanes; (**b**) SST time-series for extratropical cyclones; (**c**) SST time-series for the Mediterranean tropical storms; (**d**) SST time-series for the Mediterranean tropical depressions and Disturbances.

The SST time series also highlighted the onset of thermal drops a few days before the early stages of Mediterranean hurricane formation (Fig. 4a). In contrast, Mediterranean tropical storms and extra-tropical cyclones exhibited thermal drops slightly later, mostly during the early phases of the cyclone’s lifetimes (Figs. 4b,c). On the other hand, Mediterranean tropical depression and disturbance events did not exhibit any significant thermal drops, and in some cases, the SST even increased during these events (Fig. 4d, Table S2). This is also clearly shown in the relative SST values presented in the Supplementary Material.

**Figure 5:**
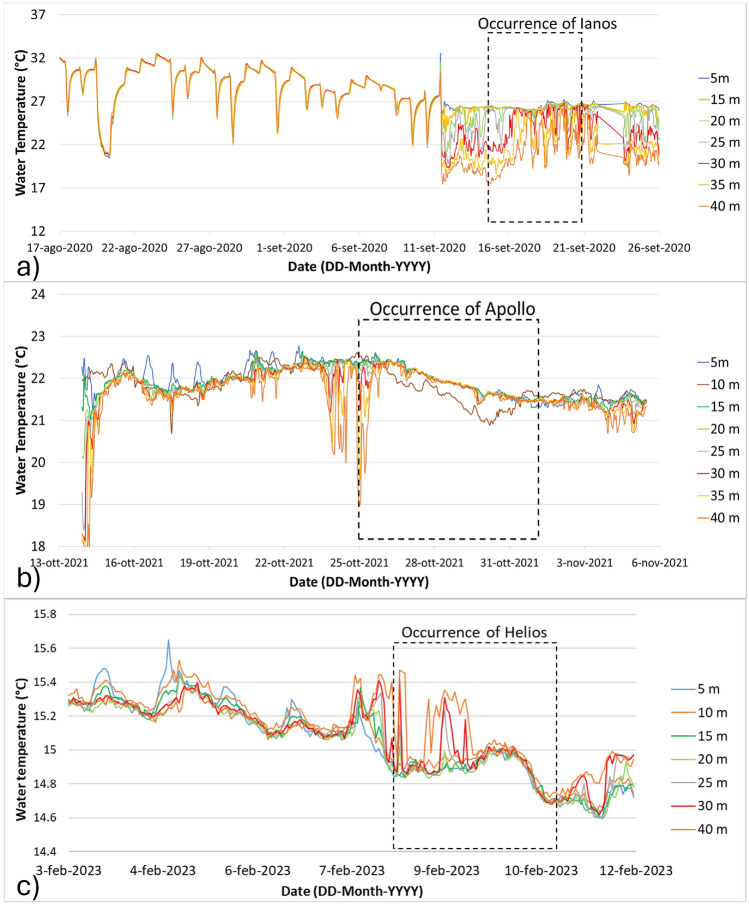
Water temperatures recorded by dataloggers installed at different water depths along Peninsula Maddalena (southeastern Sicily). (**a**) water temperatures during the occurrence of Medicane Ianos; (**b**) water temperatures during the occurrence of Medicane Apollo; (**c**) water temperatures during the occurrence of cyclone Helios.

In situ water temperatures recorded by waterproof dataloggers in southeastern Sicily highlighted the thermal drops that occurred during the Ianos, Apollo, and Helios events. Tracks of all three events affected the offshore areas close to southeastern Sicily, approximately 180 km from the coastline. The water temperature recorded at Peninsula Maddalena exhibited significant temperature decreases of 10 °C and 3.5 °C at depths of up to 40 m prior to the occurrences of Medicanes Ianos and Apollo, respectively (Fig. 5); these temperature decreases were much greater than the SST drops. In addition, the thermal drop during Medicane Ianos lasted much longer compared to Medicane Apollo. A lower decrease in SST was observed during the occurrence of the extratropical cyclone Helios^38^: this event exhibited an SST decrease of about 1.7 °C at 20 m of water depth as evaluated from an Argo float (Fig. 3).

**Figure 6:**
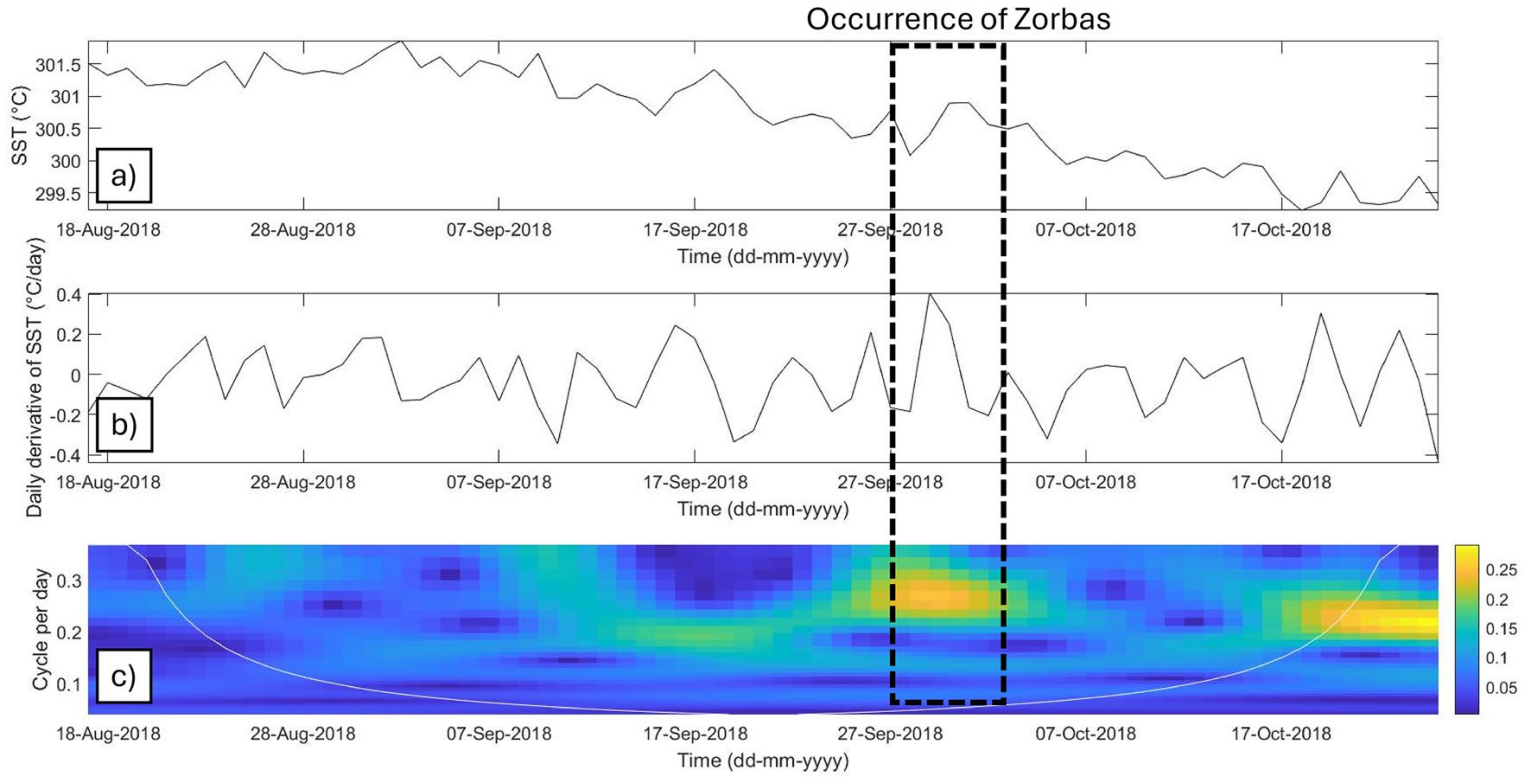
SSTs during Medicane Zorbas. (**a**) SST time-series data from ERA5; (**b**) daily derivative of SST in the analyzed time series; (**c**) CWT scalogram of the SST time series. The white line indicates the cone of influence.

**Figure 7:**
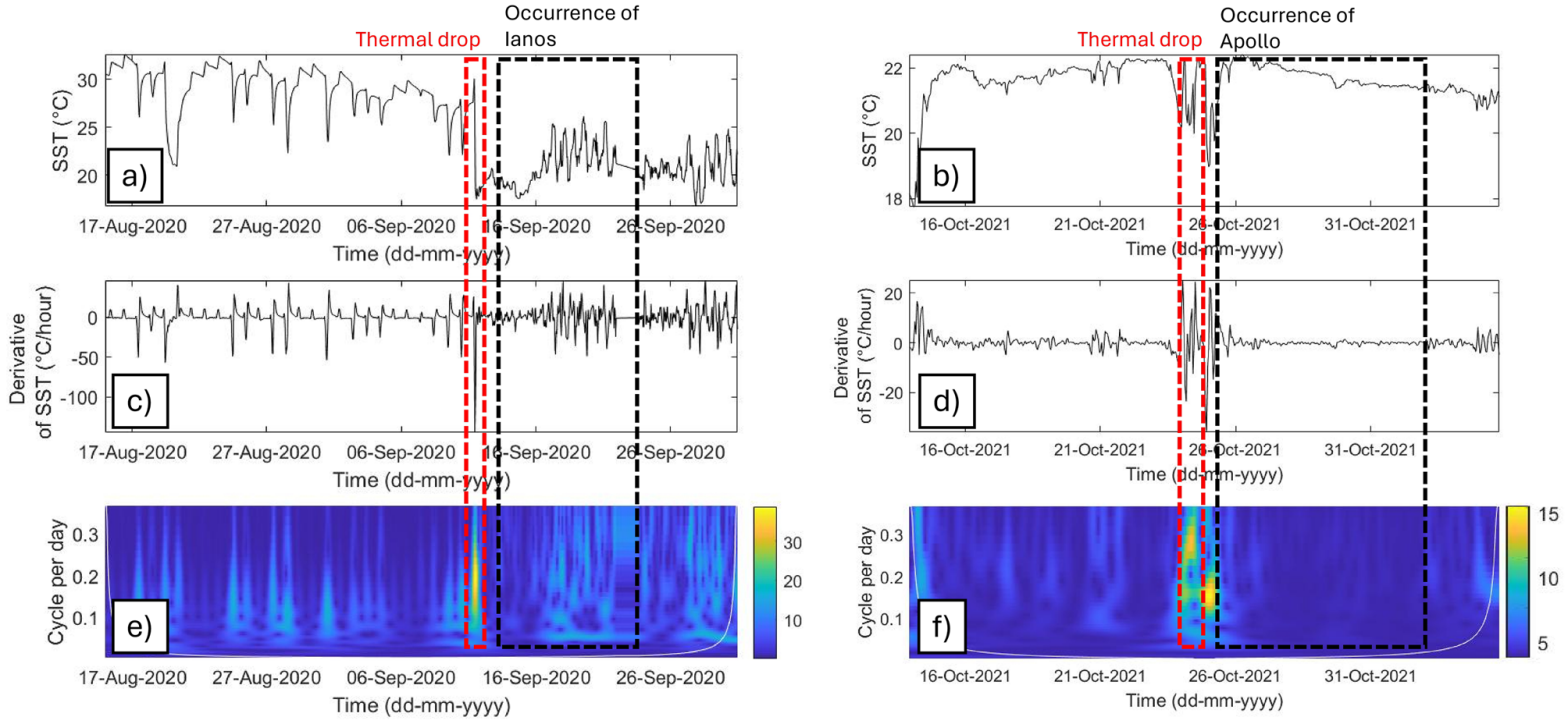
Water temperature measured by data loggers installed at a depth of 40 m. (**a**) Water temperature time-series data during Medicane Ianos. (**b**) water temperature time-series data during Medicane Apollo; (**c**) daily derivative of SST during Medicane Ianos; (**d**) daily derivative of SST during Medicane Apollo; (**e**) CWT scalogram of water temperature during Medicane Ianos; (**f**) CWT scalogram of water temperature during Medicane Apollo.

Continuous Wavelet Transform (CWT) scalograms were calculated from the SST time-series values and used to analyze the signal in the time–frequency domain. The coefficients reported in the scalogram are representative of the energy content within the SST signal at different frequencies expressed in cycles per day. The CWT scalogram of the SST revealed higher energy contents before the occurrence of Mediterranean hurricanes, with a frequency range of 0.2–0.3 cycles per day. Similar results were observed for Medicane Zorbas, which exhibited a significant increase in energy beginning 24 September 2018, with frequencies ranging from 0.2 to 0.25 cycles per day and peaking on 28 September 2018 (Fig. 6). The greatest CWT coefficients were observed for water temperatures recorded with dataloggers at a water depth of 40 m. Analyses of CWTs using data from 40 m water dataloggers highlighted the significant thermal drops during Medicanes Ianos and Apollo. The energy content was concentrated within the same range of SST frequencies of 0.2–0.3 cycles per day (Fig. 7).

## Discussion

Water temperature and SST in the Mediterranean were monitored using techniques ranging from in situ temperature data acquisition to remote sensing^36^. A notable effect of climate change in the Mediterranean region is the increase in SST anomalies^39^. SST is also an important factor in the development and intensification of Mediterranean hurricanes and extratropical cyclones^13^. Like tropical cyclones, Mediterranean hurricanes depend on latent heat and sensible heat fluxes from the sea; this is because the primary source of their energy is convection induced by the thermodynamic disequilibrium between the atmosphere and the underlying sea surface^29^. Miglietta and Rotunno^32^ and Miglietta et al.^40^ reported that intense mesoscale flows could be used to predict the occurrence of Mediterranean hurricanes. Specifically, these authors identified the occurrence of intense easterly winds over the Ionian Sea and a very strong outbreak of Tramontane and Cierzo over the western Mediterranean before cyclogenesis, respectively^32,40^. These events resulted in intense latent and sensible heat fluxes, transferring energy from the sea surface to the atmosphere.

An assessment of thermal changes using a variety of datasets revealed that satellite observations recorded higher SST values compared to Copernicus SST and ERA5 reanalysis. These features were also observed in other climate studies^41,42^, suggesting a 40% underestimate for reanalysis data compared to in situ and satellite observations. This is due to the ability of satellite observations to capture smaller scale variations (resolution of 0.01° × 0.01°), while reanalysis represents fields averaged over a wider area.

**Figure 8:**
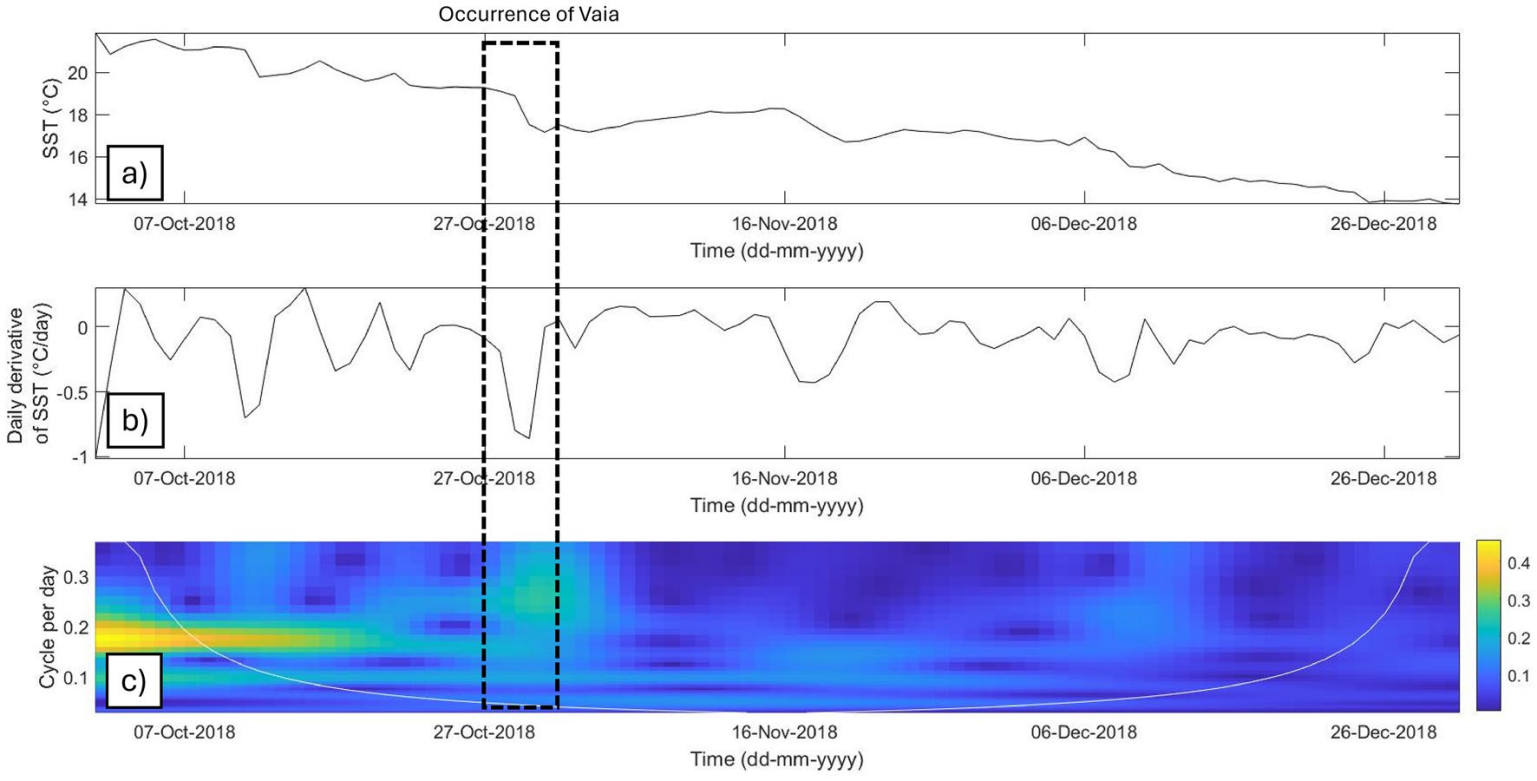
Thermal drops observed during storm Vaia (28 October 2018). (**a**) SST time series data from the storm event; (**b**) daily derivative of SST; (**c**) CWT scalogram focusing on the thermal drop during the event.

The thermal drops shown in this study were observed both before and during Mediterranean hurricane occurrences, although thermal drops during tropical cyclones are usually greater than those observed during Mediterranean hurricanes, with thermal changes in SSTs as high as 7–9 °C^43^. Bouin and Lebeaupin Brossier^44^ observed a small decrease in SSTs of − 0.18 ± 0.21 °C on 7–8 November 2014 during Medicane Qendresa. Pytharoulis^13^ and Miglietta et al.^31^ stated that SSTs play a significant role in the control and longevity of two Mediterranean tropical-like cyclones, while Noyelle et al.^45^ reported that SST has a weak influence on the tracks of cyclones but a strong influence on their intensities. We found that Medicane Ianos (15–20 September 2020) exhibited a drop in SST of about 1 °C in the location of its cyclogenesis before experiencing a secondary thermal drop of 3 °C as it moved closer to the Sicilian coast. Comparing the evolution of SST trends during Mediterranean hurricanes and Mediterranean tropical disturbances and depressions, we found that disturbances and depressions generally did not exhibit significant thermal drops, with most events exhibiting values less than 1.6 °C. Increased SSTs result in the more likely occurrence of tropical transitions, more intense lower- and upper-level warm cores, and lower pressure minima^2^. Mediterranean tropical storms and extra-tropical cyclones, such as the events that occurred in 2009, 2017, and 2018, have been associated with smaller thermal drops in the Mediterranean basin. The latter events were mainly characterized by the coupling of upper- and lower-level baroclinic processes interacting with diabatic heating to generate explosive cyclogenesis^46,47^. In particular, storm Vaia exhibited drops in SST comparable to those of Mediterranean hurricanes; however, it should be noted that the thermal drop associated with this event occurred concurrently with its formation, unlike before cyclogenesis as is the case for Mediterranean hurricanes (Fig. 8). Cavalieri et al.^48^ described the evolution of this event as explosive cyclogenesis. Spectral analysis showed that the thermal drop associated with storm Vaia occurred at a lower frequency compared to those of Mediterranean hurricane events, ranging from 0.2 to 0.3 cycles per day (Fig. 8).

A stronger change at deeper levels rather than near the sea surface was observed for tropical-like cyclones, such as Ianos (2020) and Apollo (2021); this was not observed for Helios (2023), a warm seclusion event (Fig. 5). We believe that this difference can be attributed to the strong decrease in MLD associated with the intense wind-driven upwelling induced by tropical-like cyclones (as discussed in Menna et al.^49^). In other words, the 40 m deep water at the measurement site is initially located within the mixed layer, but ends below it after the passage of the cyclone; thus, this location experiences a very strong thermal decrease. In contrast, the mechanical energy transferred from the atmosphere to the sea during Helios was weaker, and wind-driven upwelling was not as effective in producing significant changes in the sea temperature except for some small oscillations.

The sensitivity of Mediterranean hurricanes to SST was studied in the context of air-sea interaction processes by Miglietta et al.^31^, who focused on the phases that were fundamental for the development of severe convection, which is the primary mechanism that influences the sustainment of Mediterranean hurricanes. The simulations performed using the Weather Research and Forecasting (WRF) model using various SST scenarios^7,31^ revealed that the features of a Mediterranean hurricane are progressively lost as the SST decreases. In colder SST experiments, the lower intensity of the sea-surface fluxes delayed and reduced the development of convection and, consequently, cyclone intensification. In particular, the cyclone lost the characteristics of a tropical-like cyclone when the SST was reduced by more than 4 °C^44^.

Predicting the extent of increases in cyclone intensity and frequency is challenging under projected Mediterranean Sea warming scenarios^5,23,50^. However, the use of higher-resolution convection-permitting models may aid this endeavor. For example, Ricchi et al.^35^ stated that the SST distribution is fundamental to realistic simulations, and substituting recorded in situ SST value with a monthly or daily average may substantially affect the predicted intensity of simulated cyclones.

The remote sensing of SST could be integrated with other technologies, such as the microseism methods currently used for the tracking of Mediterranean hurricanes^34,51^. Microseisms represent the continuous background seismic signal caused by the interactions between the atmosphere, the hydrosphere, and the solid Earth, and are particularly useful for tracking cyclone pathways^34,52^. These techniques can be beneficial for the development of early warning systems, which are important along the Mediterranean coast where Mediterranean hurricane events can and have caused considerable damage^33,53^.

SST analyses have revealed that several Mediterranean hurricane events were preceded by thermal drops at different water depths. These thermal drops involve sharp decreases in SST values before the event, which are progressively reduced as the Mediterranean hurricane matures. Spectral analysis of these SST trends revealed that a large amount of energy was released about 3–4 days prior to Mediterranean hurricane formation, corresponding to a significant thermal drop with energy content concentrated in frequencies between 0.2 and 0.3 cycles per day. The same energy content measurements were recorded by in situ water temperature loggers at Peninsula Maddalena (southeastern Sicily), with thermal drops observed in the water column at depths of up to 40 m. The Mediterranean cyclone events with the largest observed SST drops were Ruven (November 2013), Qendresa (November 2014), Zorbas (September 2018), Trudy (November 2019), Ianos (September 2020), and Apollo (October 2021). These events were characterized by high wind speeds and MSLPs and caused a significant amount of damage to coastal areas, particularly near southeastern Sicily and Greece. The thermal drops associated with extratropical cyclones or weaker tropical-like storms had lower magnitudes compared to those of Mediterranean hurricanes. Spectral analyses of the storm events revealed low CWT coefficient values and SST decreases, which occurred almost simultaneously with the events. This thermal drop appears to be a unique feature of Mediterranean hurricanes and could be indicative of their intensities. Future projects focused on analyzing a greater amount of data and using more robust statistical methods could provide predictions with low false positive rates.

## Materials and methods

### Cyclone classification and seawater temperature data

In this study, we obtained SST data associated with the occurrence of Mediterranean cyclones using data reanalysis and satellite observations. Here, the “thermal drop” was defined as the difference between the SST 10 days prior to cyclone occurrence and at the end of the cyclone’s lifetime. We chose to measure the SST 10 days before the event to allow us to characterize temperature drops in the environment prior to the occurrence of Mediterranean hurricanes.

SST time series data from 1969 to 2023 were evaluated using satellite and reanalysis data to assess the SST changes associated with selected cyclones in the Mediterranean (a total of 52 events). A spectral analysis of the SST signals was conducted to determine the percentage of energy associated with the thermal drop during these events. The long-term SST time series were evaluated using the following datasets:

- Mediterranean Sea Physical Reanalysis (CMEMS MED-Currents Copernicus Marine Environment Monitoring Service (CMEMS), product^54^ available from 1987 to 2023
- European Centre for Medium-Range Weather Forecasts (ECMWF) reanalysis v5 (ERA5)^55^ from Copernicus Climate Change Service (C3S): SST values extracted from 1969 to 1985
- Satellite data observation from CMEMS Reprocessed (REP) Mediterranean (MED) dataset: Level 4 (L4)^56^ available from 1982 to 2023.
- Data from the International Argo program, part of the Global Ocean Observing System^57^.

MSLP and wind components at 10 m above the sea level were extracted from the ERA5 model in order to identify the cyclone events with significant MSLPs and wind speeds; the results are reported in Table S1.

Wind data from ERA5 were extracted in the following two components: the eastward wind component (U wind) and the northward wind component (V wind). These components were combined to obtain the wind speed during storm events as follows (Eq. (1)):

1 $$WS=\sqrt{{U}^{2}+{V}^{2}}$$

**Figure 9:**
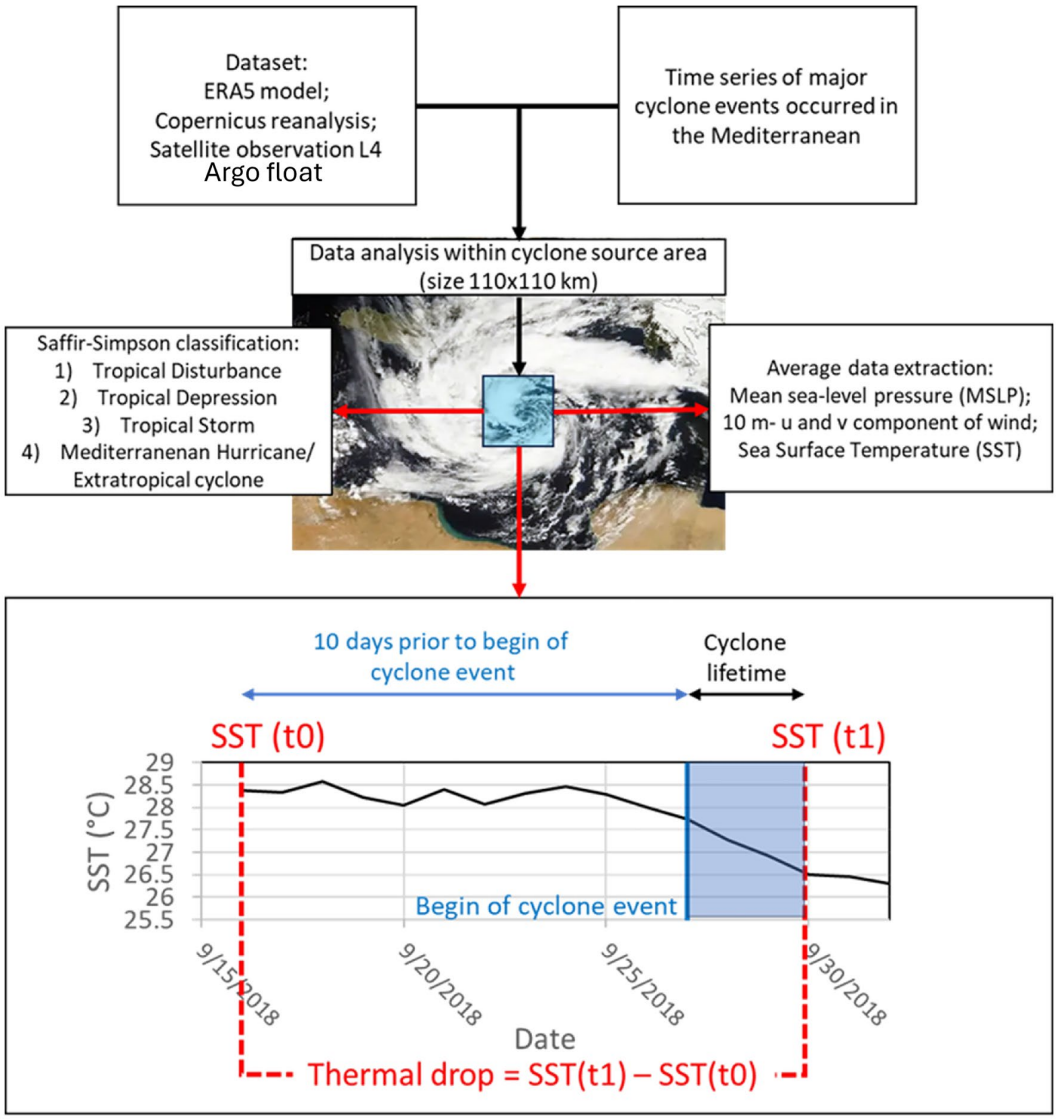
Workflow used to assess the thermal drops associated with the occurrence of selected Mediterranean cyclone events.

Daily SSTs from Copernicus reanalysis and satellite observations were used for time series SST analysis between summer and winter in years with cyclone occurrences. Time series data were extracted by resampling the cyclogenesis area (using the coordinates of cyclone formation reported in Table S1) using a 110 × 110 km grid cell (Fig. 9), within which a daily SST average was assessed. Relative SST time-series data was obtained for cyclone events by comparing the difference in SST values over a period of one day (Supplementary Material).

Vertical temperature profiles in the pre-storm and post-storm periods were extracted from Conductivity, Temperature, Depth (CTD) data of Argo floats^57^. Temperature data from the first 10 m of water was averaged and compared with SST from reanalysis and satellite data.

A spectral analysis was performed on the SST signal using a continuous wavelet transform (CWT) with the ‘Morlet’ wavelet^58^ in MatLab to identify the frequency of temperature changes for the events listed in Table S1. The SST time series were converted into daily derivatives of SST to distinguish between real periodicity and low-frequency bands in the wavelet power spectrum^59^. In this way, we highlighted pitfalls in the wavelet power spectrum^60,61^. The spectral analysis applied to the derivative of SSTs and temperature values provided the wavelet coefficients in cycles per day. The wavelet coefficients were plotted using scalograms, which represent the percentage of energy for each coefficient, with the highest energy percentage being attributed to SST changes related to Mediterranean hurricanes. The percentage of energy was divided into two frequency bands, in which frequencies less than 0.15 cycles per day represented weekly SST changes, while those greater than 0.15 cycles per day corresponded to SST changes that occurred over 10 days. The SST values from reanalysis were compared to the in situ values acquired from a series of waterproof temperature dataloggers (HOBO TidbiT MX Temperature 400), which tracked the underwater temperatures each hour with an accuracy of ± 0.2 °C. The dataloggers were installed at different depths in the Peninsula Maddalena (south-eastern Sicily, 37.0015°N, 15.3335° E) in August 2020, with a sample pass of 5 m from 5 to 40 m of water depth. The acquisition period lasted through the occurrence of the Ianos (2020) and Apollo (2021) events. In addition, a CWT analysis was performed on the water temperature signals obtained from the dataloggers to highlight the percentage of energy content along the water column during the thermal drops.

### Supplementary Information

Supplementary Information.

## Data Availability

The datasets used and/or analysed during the current study are available on the following link https://zenodo.org/record/8113525.

## References

[1] Kim G-U, Seo K-H, Chen D (2019). Climate change over the Mediterranean and current destruction of marine ecosystem. Sci. Rep..

[2] Flaounas E (2022). Mediterranean cyclones: Current knowledge and open questions on dynamics, prediction, climatology and impacts. Weather Clim. Dyn..

[3] Prezerakos N, Flocas H, Brikas D (2006). The role of the interaction between polar and subtropical jet in a case of depression rejuvenation over the eastern Mediterranean. Meteorol. Atmos. Phys..

[4] Lagouvardos K, Kotroni V, Defer E (2007). The 21–22 January 2004 explosive cyclogenesis over the Aegean Sea: Observations and model analysis. Q. J. R. Meteorol. Soc..

[5] Koseki S (2021). Modelling a tropical-like cyclone in the Mediterranean sea under present and warmer climate. Nat. Hazards Earth Sys. Sci..

[6] Farr MB (2022). An analysis of the synoptic dynamic and hydrologic character of the Black sea cyclone Falchion. Meteorology.

[7] Moscatello A, Miglietta MM, Rotunno R (2008). Observational analysis of a Mediterranean ‘hurricane’ over south-eastern Italy. Weather.

[8] Flaounas E, Gray S, Teubler F (2021). A process-based anatomy of Mediterranean cyclones: From baroclinic lows to tropical-like systems. Weather Clim. Dyn..

[9] Fita L, Romero R, Luque A, Emanuel K, Ramis C (2007). Analysis of the environments of seven Mediterranean tropical-like storms using an axisymmetric, nonhydrostatic, cloud resolving model. Nat. Hazards Earth Sys. Sci..

[10] Romera R (2017). Climate change projections of medicanes with a large multi-model ensemble of regional climate models. Glob. Planet. Change.

[11] Cavicchia L, von Storch H, Gualdi S (2014). Mediterranean tropical-like cyclones in present and future climate. J. Clim..

[12] Picornell MA, Campins J, Jansà A (2014). Detection and thermal description of medicanes from numerical simulation. Nat. Hazards Earth Sys. Sci..

[13] Pytharoulis I (2018). Analysis of a Mediterranean tropical-like cyclone and its sensitivity to the sea surface temperatures. Atmos. Res..

[14] Stathopoulos C, Patlakas P, Tsalis C, Kallos G (2020). The role of sea surface temperature forcing in the life-cycle of Mediterranean cyclones. Remote Sens..

[15] Cavicchia L, von Storch H, Gualdi S (2014). A long-term climatology of medicanes. Clim. Dyn..

[16] Gutiérrez Fernández J, Miglietta MM, González Alemán JJ, Gaertner MA. *Characteristics of Mediterranean Tropical-Like Cyclones Using ERA-5 Reanalysis*. *In Press* Geophysical Research Letters, (2024).

[17] Lionello P, Lionello P (2006). Chapter 6 cyclones in the Mediterranean region: Climatology and effects on the environment. Developments in Earth and Environmental Sciences.

[18] Lionello P (2012). The Climate of the Mediterranean Region: From the Past to the Future.

[19] Reale M (2022). Future projections of Mediterranean cyclone characteristics using the Med-CORDEX ensemble of coupled regional climate system models. Clim. Dyn..

[20] Flaounas E, Kotroni V, Lagouvardos K, Flaounas I (2014). CycloTRACK (v1.0)—tracking winter extratropical cyclones based on relative vorticity: Sensitivity to data filtering and other relevant parameters. Geosci. Model Dev..

[21] Nastos, Karavana-Papadimou K, Matsangouras IT. Tropical-like cyclones in the Mediterranean: Impacts and composite daily means and anomalies of synoptic conditions. In *Proceedings of the 14th International Conference on Environmental Science and Technology* (2015).

[22] Portmann R, González-Alemán JJ, Sprenger M, Wernli H (2020). How an uncertain short-wave perturbation on the North Atlantic wave guide affects the forecast of an intense Mediterranean cyclone (Medicane Zorbas). Weather Clim. Dyn..

[23] Miglietta MM (2019). Mediterranean tropical-like cyclones (medicanes). Atmosphere.

[24] D’Adderio LP, Casella D, Dietrich S, Sanò P, Panegrossi G (2022). GPM-CO observations of Medicane Ianos: Comparative analysis of precipitation structure between development and mature phase. Atmos. Res..

[25] McTaggart-Cowan R, Galarneau TJ, Bosart LF, Milbrandt JA (2010). Development and tropical transition of an Alpine lee cyclone part II: Orographic influence on the development pathway. Mon. Weather Rev..

[26] Buzzi A, Davolio S, Fantini M (2020). Cyclogenesis in the lee of the Alps: A review of theories. Bull. Atmos. Sci. Technol..

[27] Miglietta MM (2013). Analysis of tropical-like cyclones over the Mediterranean sea through a combined modeling and satellite approach. Geophys. Res. Lett..

[28] Tous M, Romero R (2013). Meteorological environments associated with medicane development. Int. J. Climatol..

[29] Tous M, Romero R, Ramis C (2013). Surface heat fluxes influence on medicane trajectories and intensification. Atmos. Res..

[30] Fita L, Flaounas E (2018). Medicanes as subtropical cyclones: The December 2005 case from the perspective of surface pressure tendency diagnostics and atmospheric water budget. Q. J. R. Meteorol. Soc..

[31] Miglietta M (2011). Numerical analysis of a Mediterranean ‘hurricane’ over south-eastern Italy: Sensitivity experiments to sea surface temperature. Atmos. Res..

[32] Miglietta MM, Rotunno R (2019). Development mechanisms for Mediterranean tropical-like cyclones (medicanes). Q. J. R. Meteorol. Soc..

[33] Scicchitano G (2021). Comparing impact effects of common storms and Medicanes along the coast of south-eastern Sicily. Mar. Geol..

[34] Borzì AM (2022). Monitoring extreme meteo-marine events in the Mediterranean area using the microseism (Medicane Apollo case study). Sci. Rep..

[35] Ricchi A (2019). Multi-physics ensemble versus atmosphere-ocean coupled model simulations for a tropical-like cyclone in the Mediterranean sea. Atmosphere.

[36] Shaltout M, Omstedt A (2014). Recent sea surface temperature trends and future scenarios for the Mediterranean Sea. Oceanologia.

[37] Gaertner MA (2018). Simulation of medicanes over the Mediterranean sea in a regional climate model ensemble: Impact of ocean–atmosphere coupling and increased resolution. Clim. Dyn..

[38] D’Adderio LP (2023). Helios and Juliette: Two falsely acclaimed medicanes. Atmos. Res..

[39] Masson-Delmotte V, Zhai P, Pirani A, Connors SL, Péan C, Berger S, Caud N, Chen Y, Goldfarb L, Gomis MI, Huang M, Leitzell K, Lonnoy E, Matthews JBR, Maycock TK, Waterfield T, Yelekçi O, Yu R, Zhou B, IPCC (2021). Summary for Policymakers. Climate Change 2021: The Physical Science Basis.

[40] Miglietta MM, Carnevale D, Levizzani V, Rotunno R (2021). Role of moist and dry air advection in the development of Mediterranean tropical-like cyclones (medicanes). Q. J. R. Meteorol. Soc..

[41] Zhai R, Huang C, Yang W, Tang L, Zhang W (2023). Applicability evaluation of ERA5 wind and wave reanalysis data in the south China sea. J. Oceanol. Limnol..

[42] Lavers DA, Simmons A, Vamborg F, Rodwell MJ (2022). An evaluation of ERA5 precipitation for climate monitoring. Q. J. R. Meteorol. Soc..

[43] Dare RA, McBride JL (2011). Sea surface temperature response to tropical cyclones. Mon. Weather Rev..

[44] Bouin M-N, Lebeaupin Brossier C (2020). Surface processes in the 7 November 2014 medicane from air–sea coupled high-resolution numerical modelling. Atmos. Chem. Phys..

[45] Noyelle R, Ulbrich U, Becker N, Meredith E (2018). Nat. Hazards Earth Syst. Sci. Discuss..

[46] Kouroutzoglou J (2021). Analysis of the transition of an explosive cyclone to a Mediterranean tropical-like cyclone. Atmosphere.

[47] Kouroutzoglou J (2015). On the dynamics of a case study of explosive cyclogenesis in the Mediterranean. Meteorol. Atmos. Phys..

[48] Cavaleri L, Barbariol F, Bertotti L, Besio G, Ferrari F (2022). The 29 October 2018 storm in northern Italy: Its multiple actions in the Ligurian sea. Prog. Oceanogr..

[49] Menna M (2023). A case study of impacts of an extreme weather system on the Mediterranean sea circulation features: Medicane Apollo (2021). Sci. Rep..

[50] Pytharoulis I (2018). Sensitivity of a Mediterranean tropical-like cyclone to physical parameterizations. Atmosphere.

[51] Borzì AM (2024). Long-term analysis of microseism during extreme weather events: Medicanes and common storms in the Mediterranean sea. Sci. Total Environ..

[52] Borzì A (2023). Egusphere.

[53] Scicchitano G (2020). The first video witness of Coastal Boulder displacements recorded during the impact of medicane “Zorbas” on southeastern Sicily. Water.

[54] Escudier R (2020). Mediterranean sea physical reanalysis (CMEMS MED-currents) (version 1) dataset. Copernic. Monit. Environ. Mar. Serv. (CMEMS).

[55] Yang C (2021). Sea surface temperature intercomparison in the framework of the Copernicus Climate Change Service (C3S). J. Clim..

[56] Merchant CJ (2019). Satellite-based time-series of sea-surface temperature since 1981 for climate applications. Sci. Data.

[57] Argo. *Argo Float Data and Metadata From Global Data Assembly Centre**(Argo GDAC)*. SEANOE. 10.17882/42182 (2024).

[58] Cohen MX (2019). A better way to define and describe Morlet wavelets for time-frequency analysis. NeuroImage.

[59] Hochman A, Saaroni H, Abramovich F, Alpert P (2019). Artificial detection of lower-frequency periodicity in climatic studies by wavelet analysis demonstrated on synthetic time series. J. Appl. Meteorol. Climatol..

[60] Maraun D, Kurths J (2004). Cross wavelet analysis: Significance testing and pitfalls. Nonlinear Process. Geophys..

[61] Maraun D, Kurths J, Holschneider M (2007). Nonstationary Gaussian processes in wavelet domain: Synthesis, estimation, and significance testing. Phys. Rev. E.

