# Supplementary material for: Fingerprinting Mediterranean hurricanes using pre-event thermal drops in seawater temperature
Source: Sci Rep. 2024 Apr 5;14:8014. doi: 10.1038/s41598-024-58335-w (PMC10997583; doi:10.1038/s41598-024-58335-w)
Supplement: Supplementary file 1 — Supplementary Information. [file 41598_2024_58335_MOESM1_ESM.docx]

Supplementary Materials

**Table S1.** Major cyclones observed in the Mediterranean basin. Cyclones were categorised according to one of the following groups based on ERA 5 dataset for MLSP and wind speed: Mediterranean Tropical Disturbances, Mediterranean Tropical Depressions, Mediterranean Tropical Storms, Mediterranean Hurricane and Extratropical Cyclones.

| **Event** | **Date of occurrence** | **Location of cyclone formation** | **MSLP (hPa)** | **Wind speed ERA 5 (km/h)** | **Classification** | **Reference** |
| --- | --- | --- | --- | --- | --- | --- |
| Severe Medistorm 01M | 11-13 September 1969 | Lat 39.52 – Long 12.31 E | 1006 | 72.4 | Mediterranean Tropical Depression | ^1^ |
| Caecilia 1969 | 22-27 September 1969 | Lat 35.42 – Long 18.18 E | 1001 | 80 | Mediterranean Hurricane | ^1^ |
| Sofia 1976 | 18-20 August 1976 | Lat 45.07 – Long 13.07 E | 1013 | 51.6 | Mediterranean Tropical Storm | ^2^ |
| Leucosia 1982 | 24-28 January 1982 | Lat 33.63 - Long 13.34 E | 994 | 76.4 | Mediterranean Hurricane | ^2,3^ |
| Callisto 1983 | 27 September-2 October 1983 | Lat 38.31 – Long 11.76 E | 1003 | 70.6 | Mediterranean Hurricane | ^3^ |
| Terek 1985 | 17-18 September 1985 | Lat 37.37 – Long 10.81 E | 1009 | 46.2 | Mediterranean Tropical Storm | ^2^ |
| Francisca 1985 | 27-29 October 1985 | Lat 38.49 – Long 8.33 E | 1006 | 50.4 | Mediterranean Tropical Storm | ^2^ |
| Maximus 1985 | 13-18 December 1985 | Lat 35.5 – Long 17.6 E | 1010 |  | Mediterranean Hurricane | ^4^ |
| Celeno 1995 | 14-18 January 1995 | Lat 35 – Long 15 E | 989 | 87.4 | Mediterranean Hurricane | ^5–7^ |
| Amada 1996 | 11-13 September 1996 | Lat 38.56 – Long 0.68 E | 995 |  | Mediterranean Tropical Storm | ^2^ |
| Samir 1996 | 4-6 October 1996 | Lat 36.82 – Long 12.24 E | 1001 |  | Mediterranean Tropical Storm | ^2^ |
| Cornelia 1996 | 6-11 October 1996 | Lat 40 – Long 10.3 E | 1000 | 74 | Mediterranean Hurricane | ^8^ |
| Zeo 2005 | 12-15 December 2005 | Lat 34.58 - Long 17.30 E | 989 | 93.5 | Mediterranean Hurricane | ^9,10^ |
| Akle 2011 | 02-03 January 2011 | Lat 33.38-Long 17.20 E | 1005 | 60 | Mediterranean Tropical Storm | ^2^ |
| Crisante 2011 | 01-02 April 2011 | Lat 33.94 - Long 9.47 E | 1008 | 48.8 | Mediterranean Tropical Depression | ^2^ |
| Disturbance 2011 | 04-05 July 2011 | Lat 41.55 - Long 6.61 E | 1009 | 55.8 | Mediterranean Tropical Disturbance | ^2^ |
| Nasamons 2011 | 28-30 September 2011 | Lat 34.49 – Long 16.69 E | 1009 | 50 | Mediterranean Tropical Storm | ^2^ |
| Rolph 2011 | 6-9 November 2011 | Lat 37.80 - Long 6.60 E | 996 | 71 | Mediterranean Tropical Storm/Mediterranean Hurricane | ^11,12^ |
| Athos 2012 | 09-12 March 2012 | Lat 28.81 – Long 10.23 E | 996 | 72 | Mediterranean Tropical Storm | ^2^ |
| Lucia 2012 | 13-15 April 2012 | Lat 38.85 – Long 11.23 E | 982 | 70 | Mediterranean Tropical Storm | ^2^ |
| Honorata 2012 | 16-17 April 2012 | Lat 42.47 - Long 17.61 E | 998 | 60 | Mediterranean Tropical Depression | ^2^ |
| Cristina 2012 | 03-06 September 2012 | Lat 41.20 – Long 13.21 E | 1006 | 55 | Mediterranean Tropical Depression | ^2^ |
| Zuetina 2012 | 29-Dec 2012 – 02 Jan 2013 | Lat 33.41 – Long 21.32 E | 1009 | 50 | Mediterranean Tropical Depression | ^2^ |
| Ruven 2013 | 17-19 November 2013 | Lat 41.75 – Long 4.57 E | 990 | 71 | Mediterranean Tropical Storm | ^13^ |
| Ilona 2014 | 19 January 2014 | Lat 41.75 – Long 4.57 E | 988 | 90.5 | Mediterranean Tropical Storm | ^13^ |
| Qendresa 2014 | 7-9 November 2014 | Lat 36.22 - Long 12.03 E | 993 | 87.6 | Mediterranean Hurricane | ^13,14^ |
| Xandra 2014 | 1-4 December 2014 | Lat 37.16- Long 1.94 E | 990 | 76 | Mediterranean Tropical Storm | ^13^ |
| Storm 2014 | 18-19 June 2014 | Lat 41.62 – Long 3.70 E | 1013 |  | Mediterranean Tropical Disturbance | ^2^ |
| Anton 2015 | 3-7 March 2015 | Lat 41.42 – Long 9.88 E | 996 | 69 | Mediterranean Tropical Storm | ^15^ |
| Berardo 2015 | 20-21 March 2015 | Lat 36.13 – Long -3.49 E | 1001 | 60 | Mediterranean Tropical Depression | ^2^ |
| Erik 2015 | 21-22 May 2015 | Lat 43.51 – Long 8.48 E | 1003 | 55 | Mediterranean Tropical Depression | ^2^ |
| Isabella 2015 | 09-11 September 2015 | Lat 37.36 – Long 5.85 E | 1003 | 47 | Mediterranean Tropical Storm | ^2^ |
| Messala 2015 | 30 September – 02 October 2015 | Lat 38.78 – Long 3.77 E | 1007 | 61 | Mediterranean Tropical Storm | ^15^ |
| Requiario 2015 | 19-20 October 2015 | Lat 36.81 – Long -0.05 E | 1006 | 48 | Mediterranean Tropical Depression | ^2^ |
| Zissi 2016 | 28 Febrary – 02 March 2016 | Lat 41.36 – Long 8.56 E | 987 | 74 | Mediterranean Tropical Storm | ^2^ |
| Caulonia 2016 | 16-17 March 2016 | Lat 37.57 – Long 15.81 E | 1007 | 59 | Mediterranean Tropical Depression | ^2^ |
| Florius 2016 | 22-25 June 2016 | Lat 37.06 – Long 13.61 E | 1011 | 20 | Mediterranean Tropical Depression | ^2^ |
| Jehanne 2016 | 23-25 July 2016 | Lat 42.28 – Long 7.34 E | 1008 | 52 | Mediterranean Tropical Depression | ^2^ |
| Krimisa 2016 | 08-11 September 2016 | Lat 38.15 – Long 12.12 E | 1005 | 50 | Mediterranean Tropical Depression | ^2^ |
| 2016 | 20-21 October 2016 | Lat 38.08 – Long 3.04 E | 1008 | 47 | Mediterranean Tropical Depression | ^2^ |
| Trixie 2016 | 28 October-1 November 2016 | Lat 38.05 - Long 17.92 E | 1010 | 70 | Mediterranean Tropical Storm | ^10,16,17^ |
| Numa 2017 | 15-19 November 2017 | Lat 40.09 - Long 14.31 E | 1004 | 66 | Mediterranean Tropical Storm | ^16,18^ |
| Zorbas 2018 | 27-30 September 2018 | Lat 32.79 - Long 20.35 E | 995 | 88 | Mediterranean Hurricane | ^10,19–22^ |
| Vaia 2018 | 27-30 October 2018 | Lat 43.17 – Long 7.78 E | 979 | 100 | Extratropical cyclone | ^23,24^ |
| Storm 2019 | 01-02 January 2019 | Lat 37.29 – Long 16.46 E | 1004 | 69 | Mediterranean Tropical Storm | ^2^ |
| Depression 2019 | 09-14 April 2019 | Lat 41.62 – Long 7.21 E | 1003 | 45 | Mediterranean Tropical Depression | ^2^ |
| Trudy 2019 | 11-13 November 2019 | Lat 37.80 - Long 6.60 E | 987 | 78 | Mediterranean Hurricane | ^16^ |
| Ianos 2020 | 15-20 September 2020 | Lat 34.68 - Long 18.40 E | 1000 | 64 | Mediterranean Hurricane | ^17,25^ |
| Depression 2021 | 14-15 February 2021 | Lat 32.70 – Long 20.18 E | 1011 | 61 | Mediterranean Tropical Depression | ^2^ |
| Disturbance 2021 | 11-12 September 2021 | Lat 39.82 – Long 7.84 E | 1014 | 37 | Tropical Disturbance | ^2^ |
| Apollo 2021 | 25 October-1 November 2021 | Lat 34.10 - Long 15.94 E | 1003 | 66 | Mediterranean Hurricane | ^26,27^ |
| Blas 2021 | 06-18 November 2021 | Lat 38.62 – Long 3.02 E | 1005 | 75 | Extratropical cyclone | ^2^ |
| Ciprian 2022 | 16-20 October 2022 | Lat 34.45 – Long 31.56 E | 1005 | 58 | Mediterranean Tropical Storm | ^2^ |
| Helios 2023 | 8-11 February 2023 | Lat 36 - Long 14 E | 1008 | 74 | Extratropical cyclone | ^28,29^ |
| Juliette 2023 | 28 February – 2 March 2023 | Lat 40 – Long 5.28 E | 997 | 80 | Extratropical cyclone | ^29^ |
| Daniel 2023 | 10-12 September 2023 | Lat 34.23 – Long 14.99 E | No Data |  | Medicane/Extratropical cyclone | Extracted from L3S-L4 satellite observations^59^ |

**Table S2.** Thermal drop assessed from different dataset for the Mediterranean Tropical Depression, Mediterranean Tropical Storm, Mediterranean Hurricanes, and Extratropical cyclones with CWT range.

| EVENTS | Thermal drop assessed from different dataset | | | | Classification | CWT |
| --- | --- | --- | --- | --- | --- | --- |
|  | Satellite L4 | Reanalysis | ERA5 | Argo float |  |  |
| Medstorm 1969 |  |  | 0.35 | 1.236 | Mediterranean Tropical Depression | 0.05-0.1 |
| Caecilia 1969 |  |  | -1.22 | 1.4385 | Mediterranean Hurricane | 0.1-0.2 |
| Sofia 1979 |  |  | 0.22 | 0.422625 | Mediterranean Tropical Storm | 0.05-0.1 |
| Leucosia 1982 | -0.3 |  | -0.16 |  | Mediterranean Hurricane | 0.1-0.2 |
| Callisto 1983 |  |  | -1.83 |  | Mediterranean Hurricane | 0.05-0.1 |
| Francisca 1985 |  | -1.54 |  | -0.236 | Mediterranean Tropical Storm | 0.05-0.1 |
| Maximus 1985 |  | -1.09 |  | -3.081 | Mediterranean Hurricane | 0.05-0.1 |
| Celeno 1995 | -1.49 | -1.06 | -1.24 | -1.684 | Mediterranean Hurricane | 0.2-0.25 |
| Cornelia 1996 | -0.78 | -0.97 | -1.37 | -0.09939 | Mediterranean Hurricane | 0.2-0.25 |
| Zeo 2005 | -1.62 | -1.12 | -1.21 | 1.110833 | Mediterranean Hurricane | 0.1-0.2 |
| Akle 2011 | -0.21 |  |  | -0.4805 | Mediterranean Tropical Storm | 0.1-0.15 |
| Crisante 2011 | 0.88 |  |  | 0.0885 | Mediterranean Tropical Depression | 0.05-0.1 |
| Tropical Disturbance 2011 | 1.16 |  |  | 0.39225 | Mediterranean Tropical Disturbance | 0.05-0.15 |
| Nasamons 2011 | -1.48 |  |  | 0.207 | Mediterranean Tropical Storm | 0.05-0.15 |
| Rolph 2011 | -1.81 | -1.48 | -1.88 | 0.2505 | Mediterranean Tropical Storm | 0.1-0.2 |
| Athos 2012 | 0.07 |  |  | 0.66025 | Mediterranean Tropical Storm | 0.05-0.15 |
| Lucia 2012 | -0.43 |  |  | 0.471 | Mediterranean Tropical Storm | 0.05-0.15 |
| Honorata 2012 | 0.74 |  |  |  | Mediterranean Tropical Depression | 0.05-0.15 |
| Cristina 2012 | -2.94 |  |  |  | Mediterranean Tropical Storm | 0.05-0.15 |
| Zuetina 2012 | -0.81 |  |  | -0.182 | Mediterranean Tropical Depression | 0.05-0.15 |
| Ruven 2013 | -2.83 | -2.40 | -2.74 | -1.794 | Mediterranean Hurricane | 0.1-0.2 |
| Illona 2014 | 0.40 | -0.36 | -0.25 |  | Mediterranean Tropical Storm | 0.1-0.2 |
| Qendresa 2014 | -1.91 | -1.46 | -1.50 |  | Mediterranean Hurricane | 0.3-0.4 |
| Xandra 2014 | -0.98 | -1.00 | -1.18 | -0.127 | Mediterranean Tropical Storm | 0.3-0.4 |
| Tropical Disturbance 2014 | 2.07 |  |  | -1.126 | Mediterranean Tropical Disturbance | 0.1-0.2 |
| Anton 2015 | -0.03 |  |  | 0.066514 | Mediterranean Tropical Storm | 0.1-0.2 |
| Berardo 2015 | 0.26 |  |  | -1.27425 | Mediterranean Tropical Depression | 0.1-0.2 |
| Erik 2015 | -1.01 |  |  | -0.092 | Mediterranean Tropical Depression | 0.1-0.2 |
| Isabella 2015 | -0.5 |  |  | -1.2135 | Mediterranean Tropical Storm | 0.1-0.2 |
| Messala 2015 | -1.7 |  |  | -2.0335 | Mediterranean Tropical Storm | 0.2-0.25 |
| Requiario 2015 | -0.58 |  |  | -0.32803 | Mediterranean Tropical Depression | 0.1-0.2 |
| Zissi 2016 | -0.26 |  |  | -1.0585 | Mediterranean Tropical Storm | 0.1-0.2 |
| Caulonia 2016 | 0.49 |  |  | -0.9932 | Mediterranean Tropical Depression | 0.05-0.15 |
| Florius 2016 | 0.08 |  |  | -1.95189 | Mediterranean Tropical Depression | 0.05-0.15 |
| Jehannie 2016 | 1.11 |  |  | -1.32225 | Mediterranean Tropical Depression | 0.05-0.15 |
| Krimisa 2016 | -0.64 |  |  |  | Mediterranean Tropical Depression | 0.1-0.2 |
| Trixie 2016 | -1.58 | -1.60 | -1.52 |  | Mediterranean Tropical Storm | 0.2-0.25 |
| Numa 2017 | -1.55 | -1.58 | -1.34 |  | Mediterranean Tropical Storm | 0.2-0.25 |
| Zorbas 2018 | -3.00 | -2.33 | -2.28 |  | Mediterranean Hurricane | 0.2-0.25 |
| Vaia 2018 | -1.31 | -1.18 | -1.04 |  | Extratropical cyclone | 0.05-0.2 |
| Tropical Storm 2019 | -1.06 |  |  |  | Mediterranean Tropical Storm | 0.1-0.2 |
| Tropical Depression 2019 | -0.09 |  |  | -0.698 | Mediterranean Tropical Depression | 0.1-0.2 |
| Trudy 2019 | -2.73 | -2.55 | -2.49 | -1.4775 | Mediterranean Hurricane | 0.1-0.2 |
| Ianos 2020 | -1.00 | -1.64 | -1.57 | -2.84075 | Mediterranean Hurricane | 0.2-0.3 |
| Tropical Depression 2021 | -0.15 |  |  | -3.5147 | Mediterranean Tropical Depression | 0.1-0.2 |
| Tropical Disturbance 2021 | 0.05 |  |  | -1.85454 | Mediterranean Tropical Disturbance | 0.1-0.2 |
| Apollo 2021 | -2.31 | – | -1.78 | -1.7966 | Mediterranean Hurricane | 0.2-0.25 |
| Blas 2021 | -3.49 |  |  | -0.89379 | Extratropical cyclone | 0.2-0.25 |
| Ciprian 2022 | -1.83 |  |  | -4.25646 | Mediterranean Tropical Storm | 0.1-0.2 |
| Helios 2023 | -0.67 | -0.59 | -0.57 | -1.21543 | Extratropical cyclone | 0.1-0.2 |
| Juliette 2023 | -0.72 | -0.49 | -0.29 | -0.6975 | Extratropical cyclone | 0.1-0.2 |
| Daniel 2023 | -0.82 | – | – | -2.59127 | Mediterranean Hurricane/Extratropical cyclone | 0.2-0.25 |

**1.2 SST time-series for the Mediterranean cyclones**

Here, the time-series of SST and relative SST for the Mediterranean cyclone events are reported in the function of the various datasets.

| 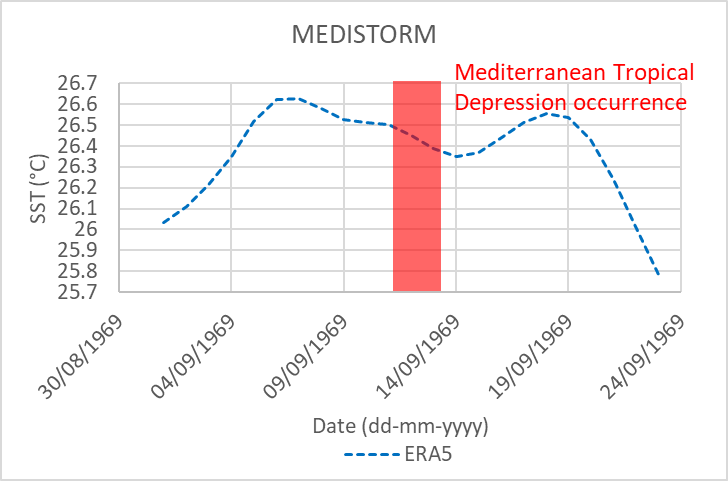  (a) | 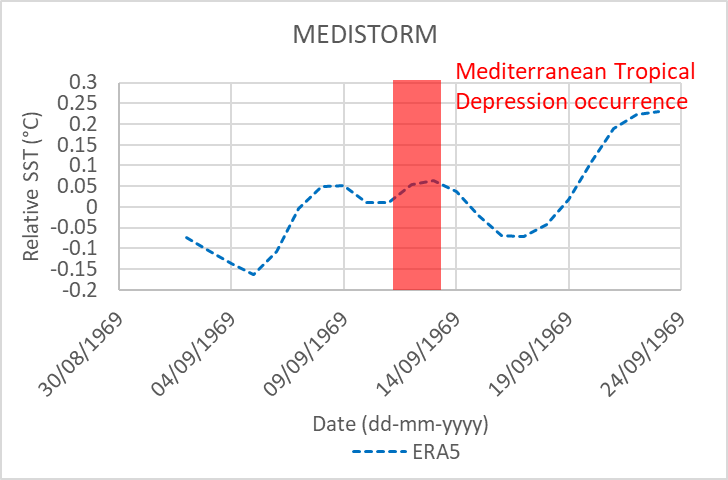  (b) |
| --- | --- |

Fig. S1 Analysis on the SST for the Mediterranean Tropical Depression MEDISTORM (11-13 September 1969) and relative occurrence reported in red bar; (a) SST time-series; (b) relative SST time-series.

| 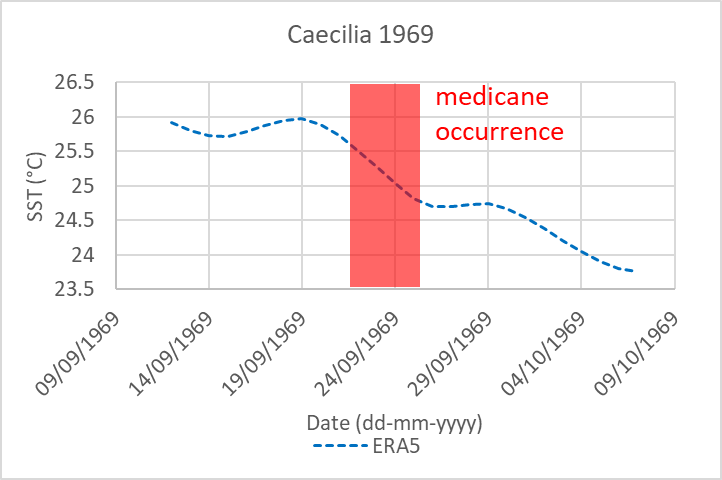  (a) | 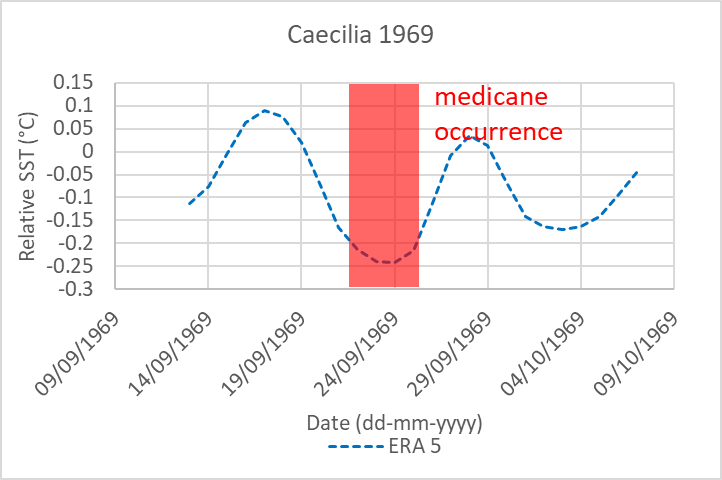  (b) |
| --- | --- |

Fig. S2 Analysis on the SST for the Medicane Caecilia(22-27 September 1969) and relative occurrence reported in red bar; (a) SST time-series; (b) relative SST time-series.

| 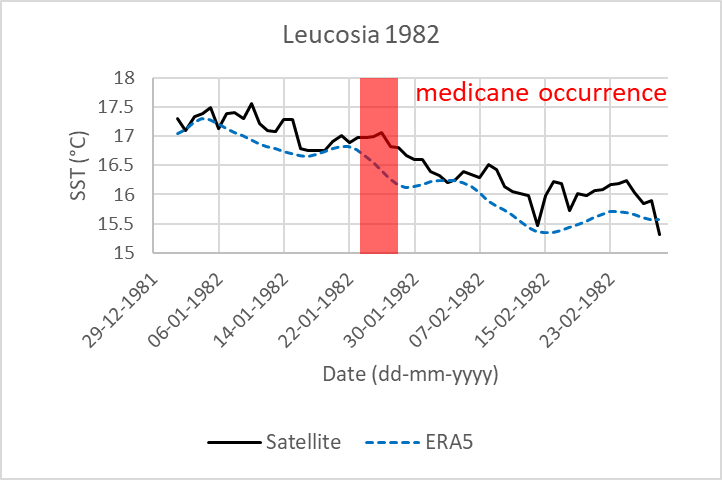  (a) | 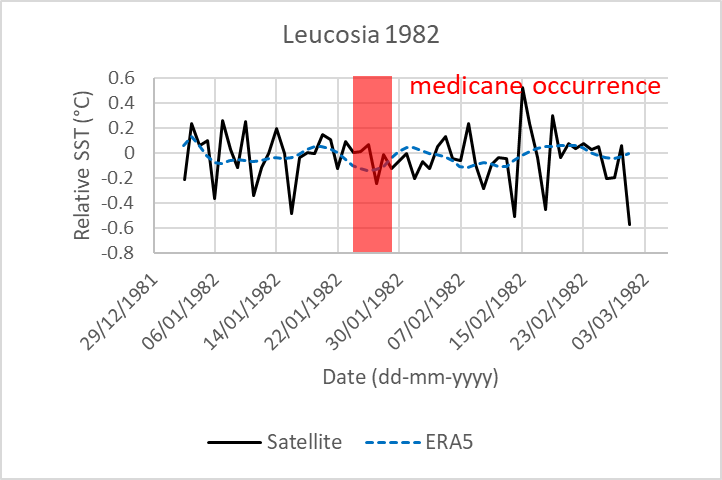  (b) |
| --- | --- |

Fig. S3 Analysis on the SST for the Medicane Leucosia (24-28 January 1982) and relative occurrence reported in red bar; (a) SST time-series; (b) relative SST time-series.

| 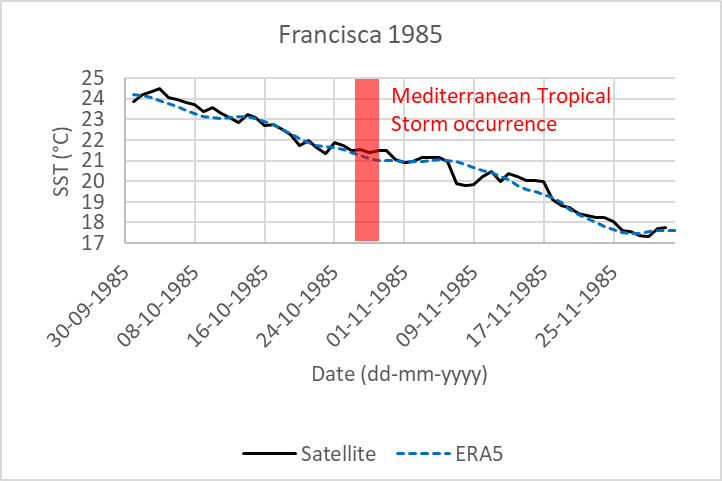  (a) | 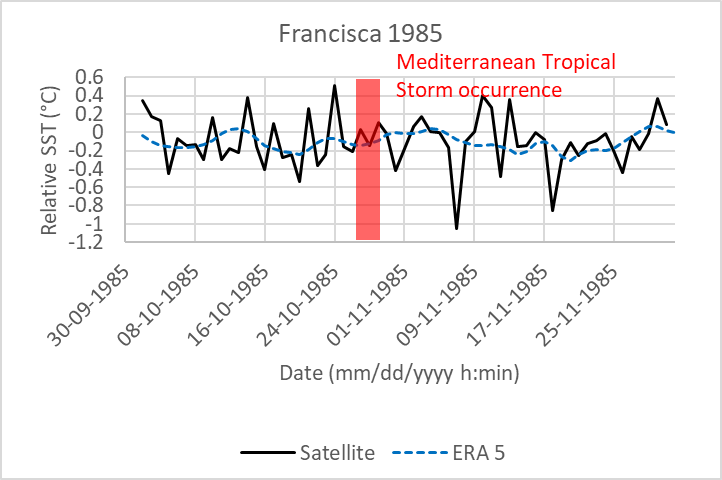  (b) |
| --- | --- |

Fig. S4 Analysis on the SST for the Francisca (27-29 October 1985) and relative occurrence reported in red bar; (a) SST time-series; (b) relative SST time-series.

| 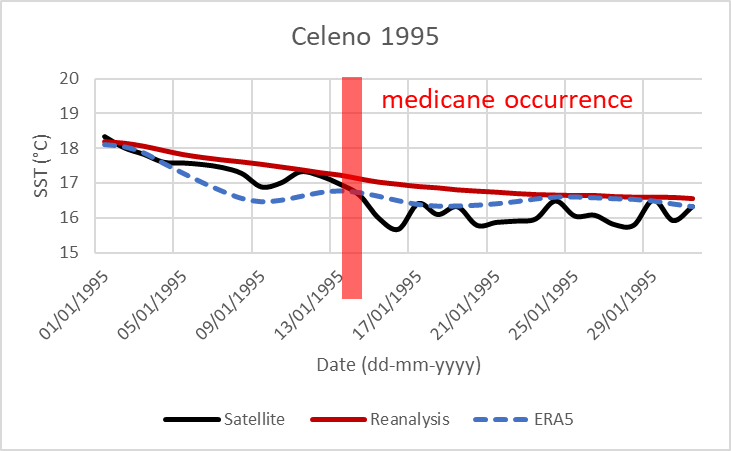  (a) | 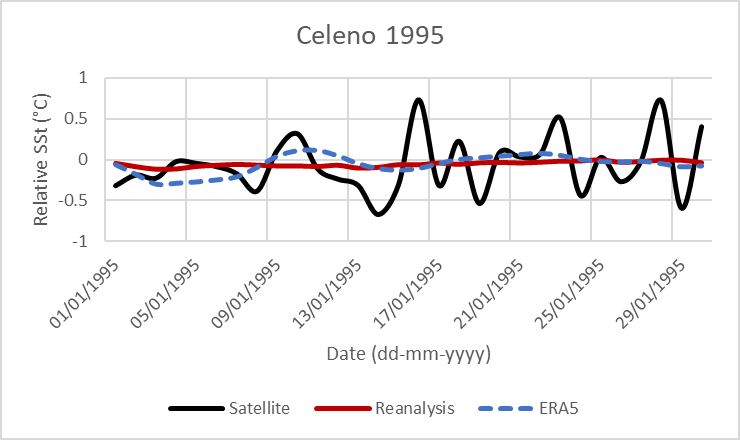  (b) |
| --- | --- |

Fig. S5 Analysis on the SST for the Medicane Celeno (14-18 January 1995) and relative occurrence reported in red bar; (a) SST time-series; (b) relative SST time-series.

| 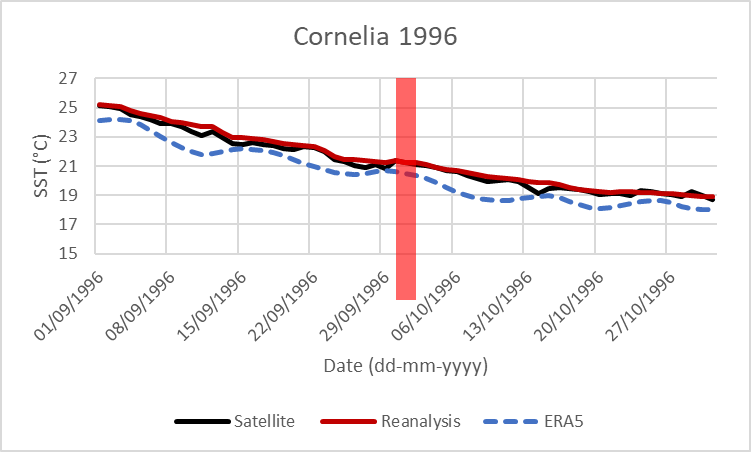  (a) | 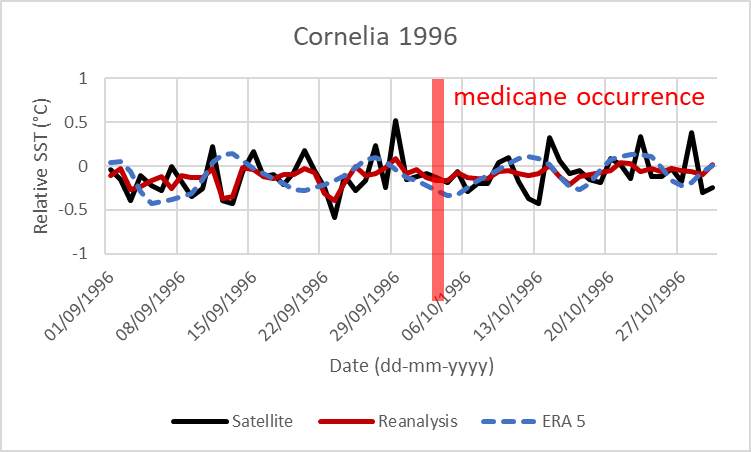  (b) |
| --- | --- |

Fig. S6 Analysis on the SST for the Medicane Cornelia (04-06 October 1996) and relative occurrence reported in red bar; (a) SST time-series; (b) relative SST time-series.

| 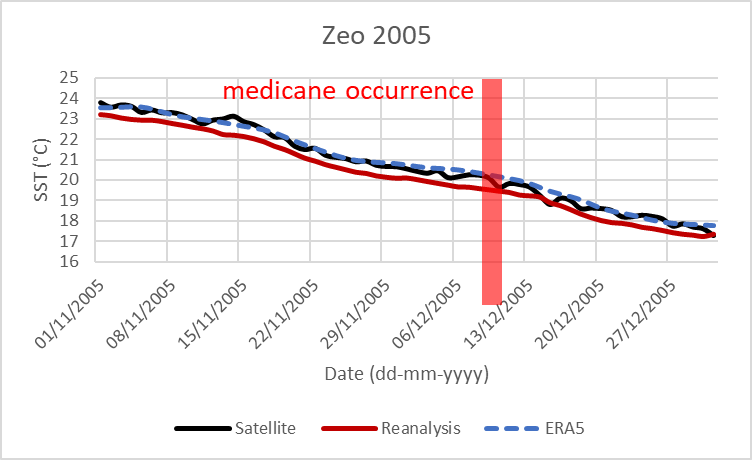  (a) | 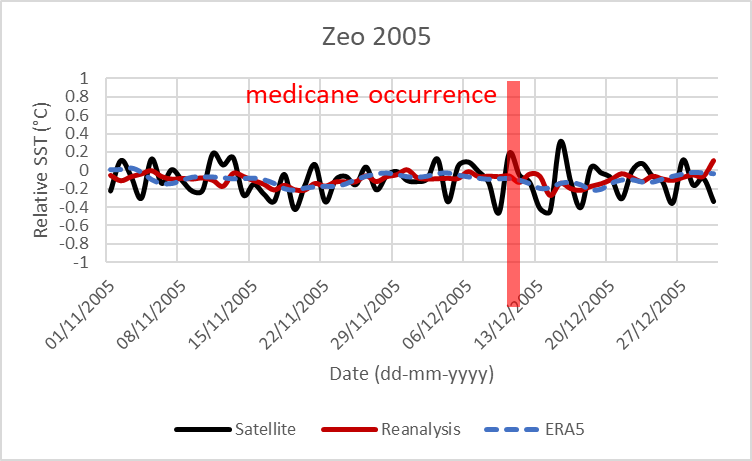  (b) |
| --- | --- |

Fig. S7 Analysis on the SST for the Medicane Zeo (12-15 October 2005) and relative occurrence reported in red bar; (a) SST time-series; (b) relative SST time-series.

| 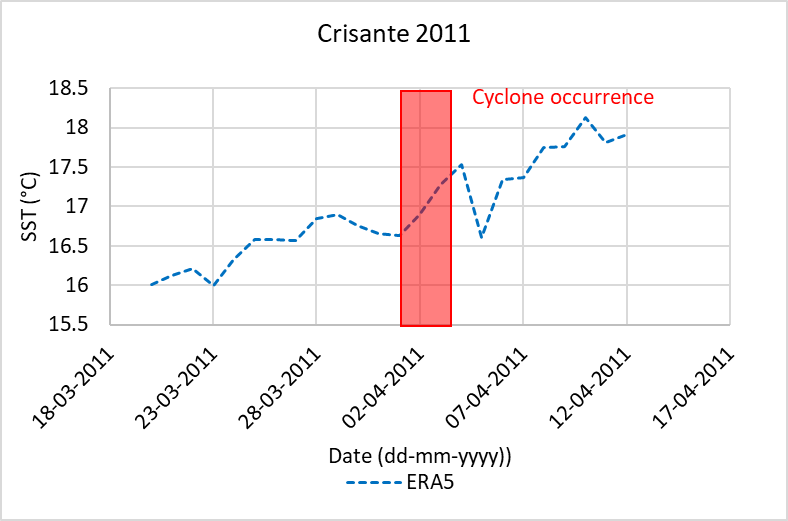  (a) | 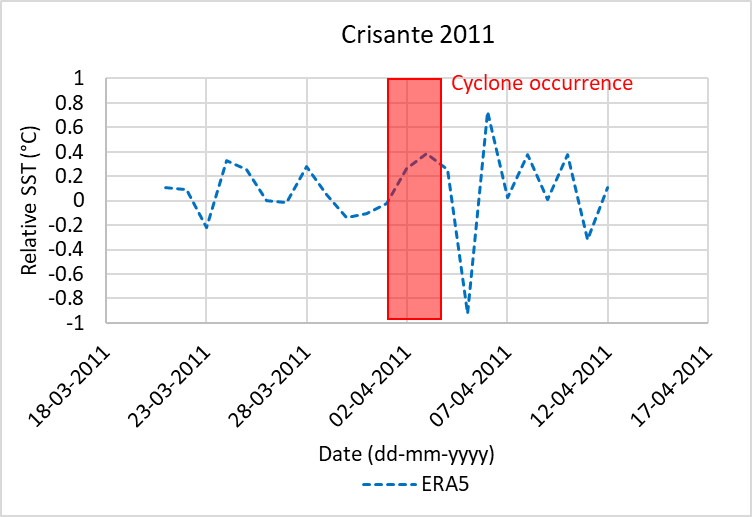  (b) |
| --- | --- |

Fig. S8 Analysis on the SST for the Crisante (02-04 April 2011) and relative occurrence reported in red bar; (a) SST time-series; (b) relative SST time-series.

| 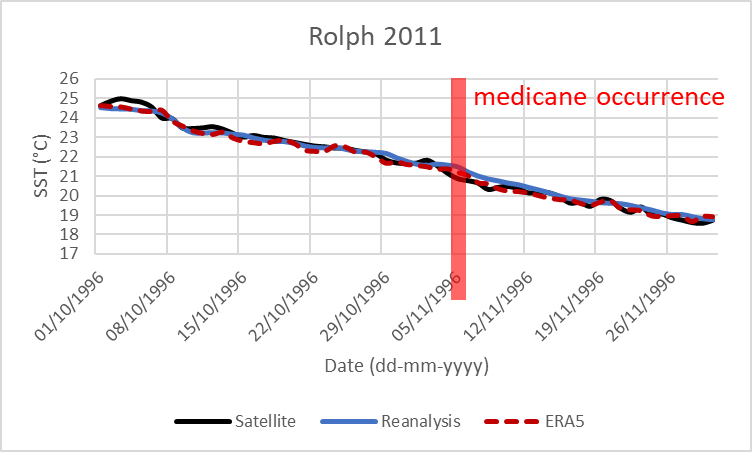  (a) | 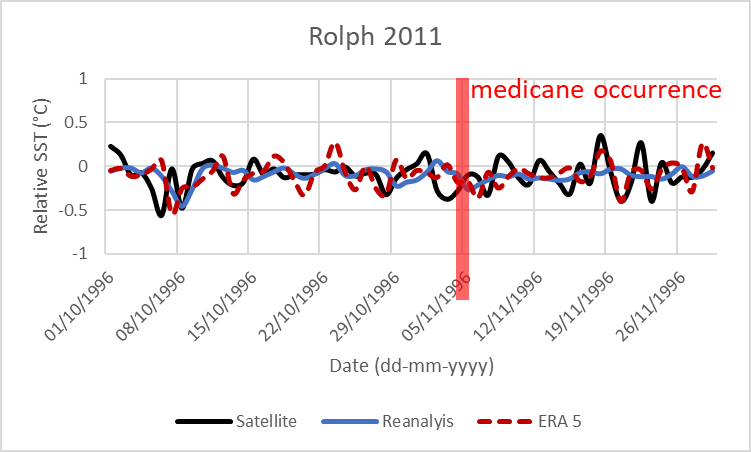  (b) |
| --- | --- |

Fig. S9 Analysis on the SST for the Medicane Rolph (06-09 November 2011) and relative occurrence reported in red bar; (a) SST time-series; (b) relative SST time-series.

| 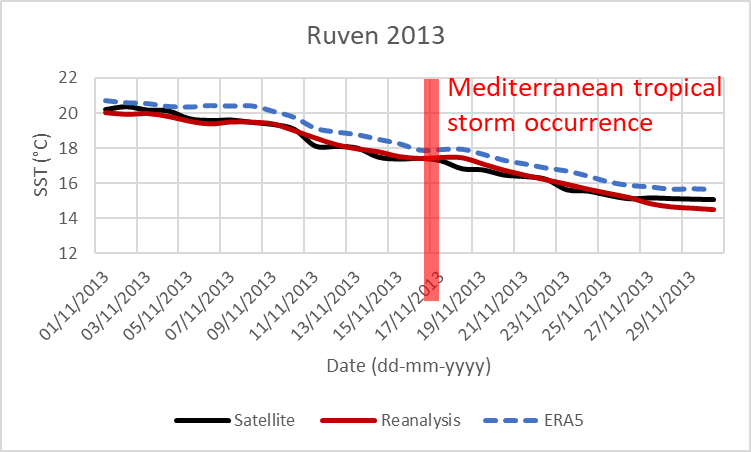  (a) | 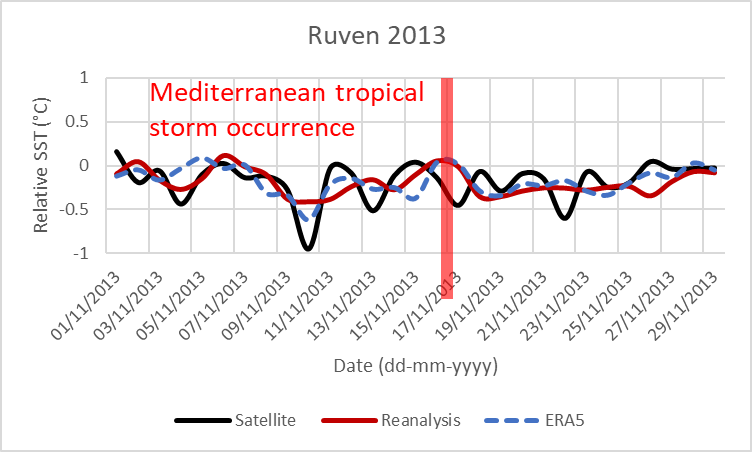  (b) |
| --- | --- |

Fig. S10 Analysis on the SST for the Medicane Ruven (17-19 November 2013) and relative occurrence reported in red bar; (a) SST time-series; (b) relative SST time-series.

| 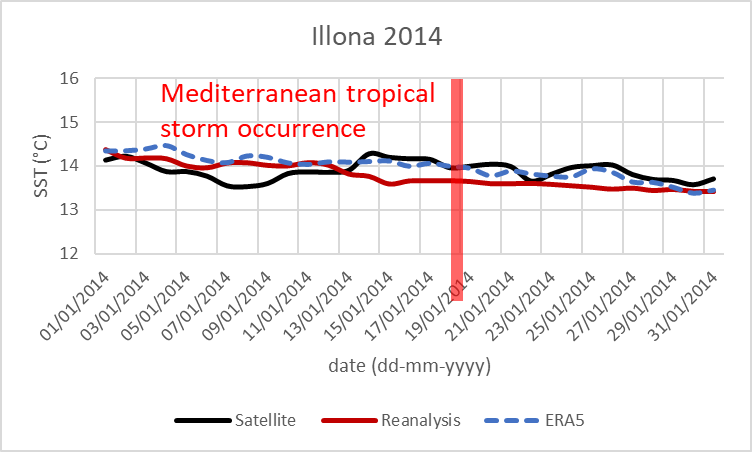  (a) | 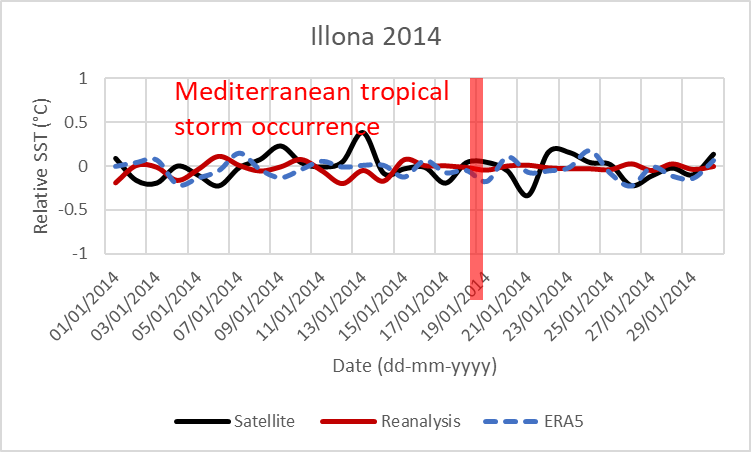  (b) |
| --- | --- |

Fig. S11 Analysis on the SST for the Mediterranean tropical storm Illona (19 January 2014) and relative occurrence reported in red bar; (a) SST time-series; (b) relative SST time-series.

| 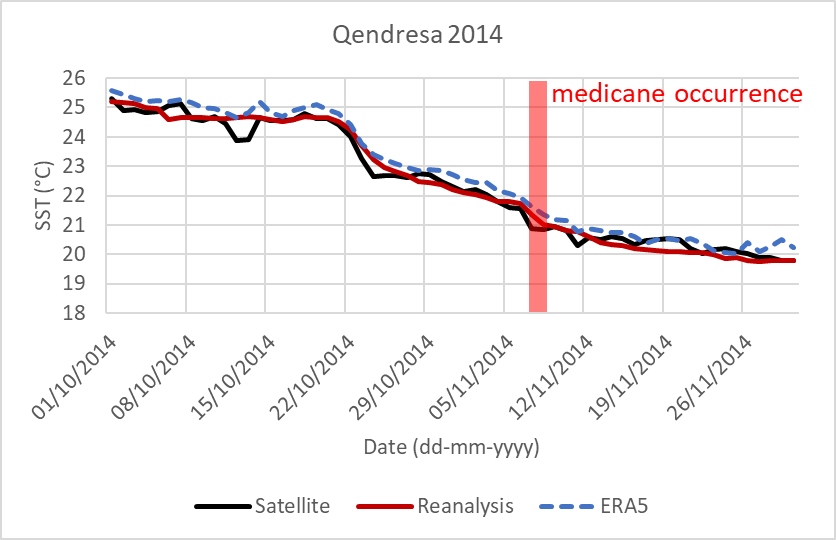  (a) | 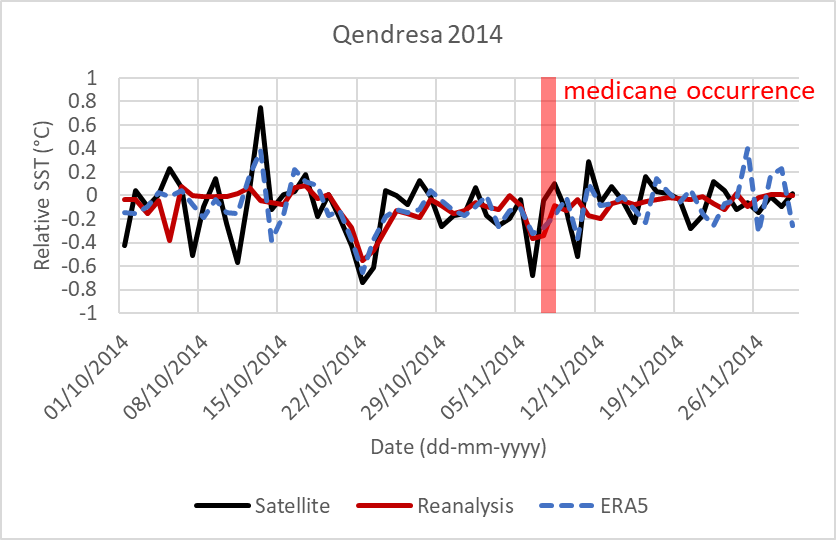  (b) |
| --- | --- |

Fig. S12 Analysis on the SST for the Medicane Qendresa (07-09 November 2014) and relative occurrence reported in red bar; (a) SST time-series; (b) relative SST time-series.

| 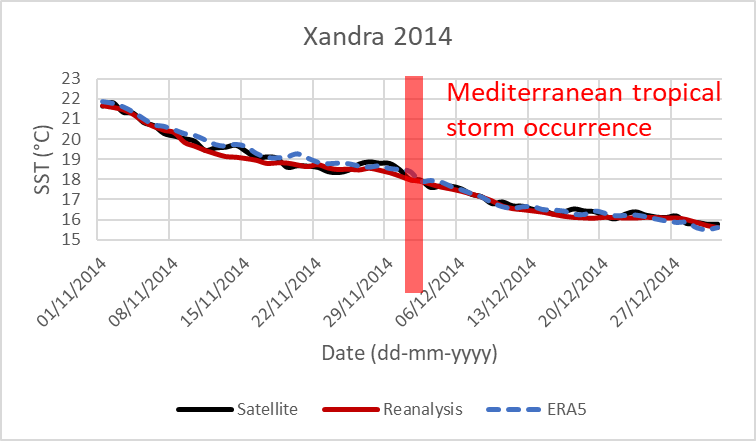  (a) | 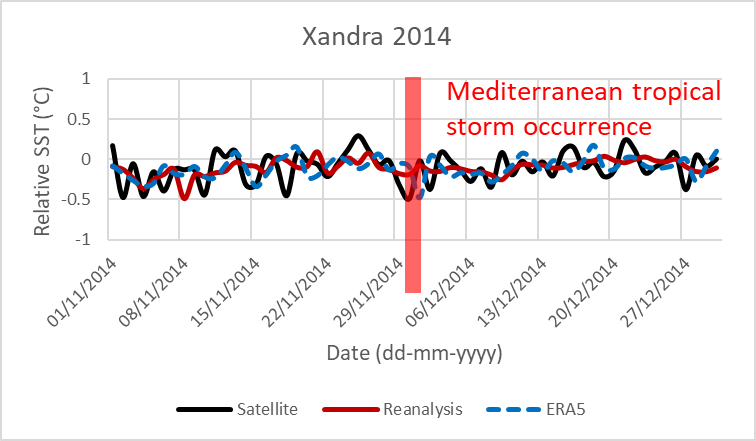  (b) |
| --- | --- |

Fig. S13 Analysis on the SST for the Mediterranean tropical storm Xandra (01-04 December 2014) and relative occurrence reported in red bar; (a) SST time-series; (b) relative SST time-series.

| 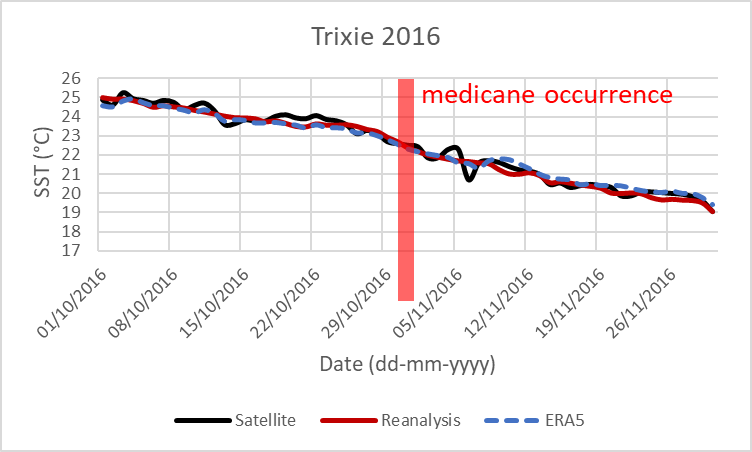  (a) | 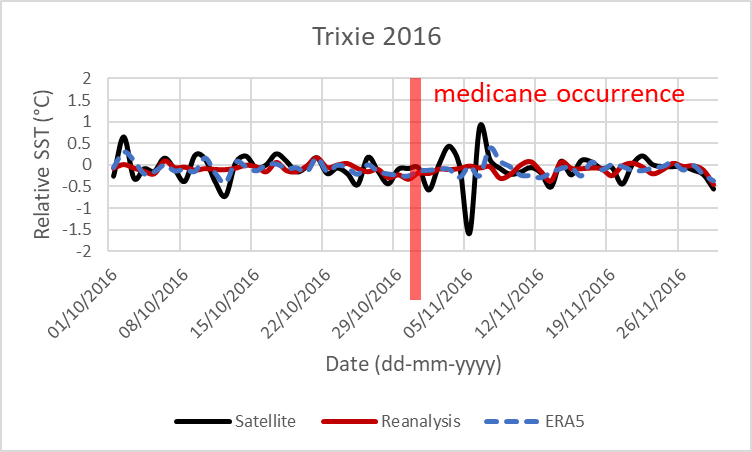  (b) |
| --- | --- |

Fig. S14 Analysis on the SST for the Medicane Trixie (28 October - 01 November 2016) and relative occurrence reported in red bar; (a) SST time-series; (b) relative SST time-series.

| 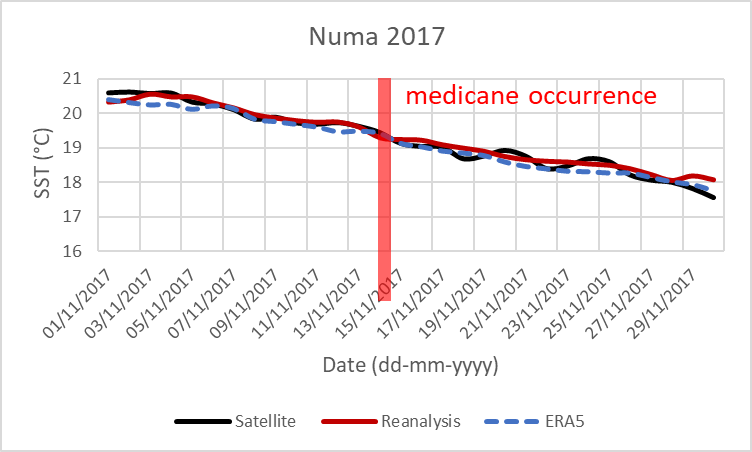  (a) | 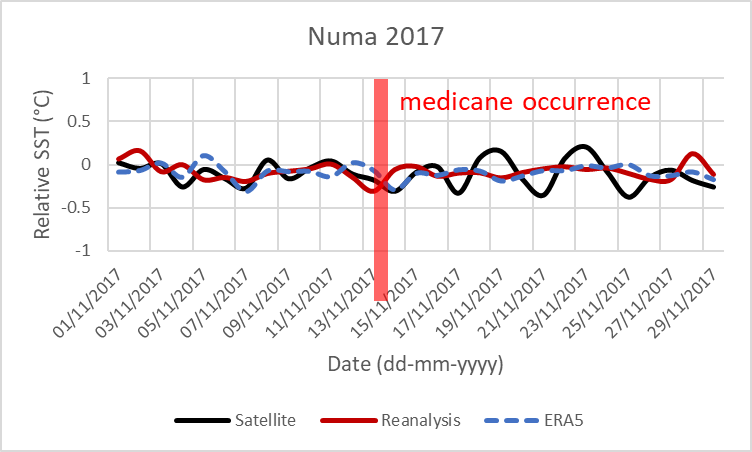  (b) |
| --- | --- |

Fig. S15 Analysis on the SST for the Medicane Numa (15-19 November 2017) and relative occurrence reported in red bar; (a) SST time-series; (b) relative SST time-series.

| 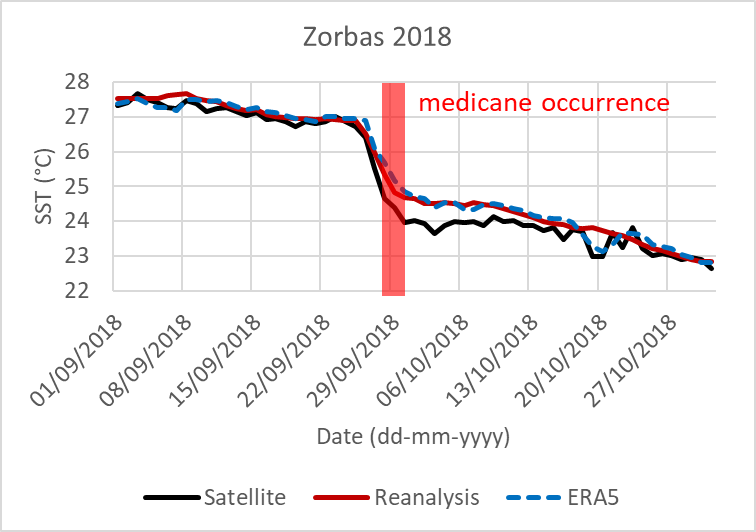  (a) | 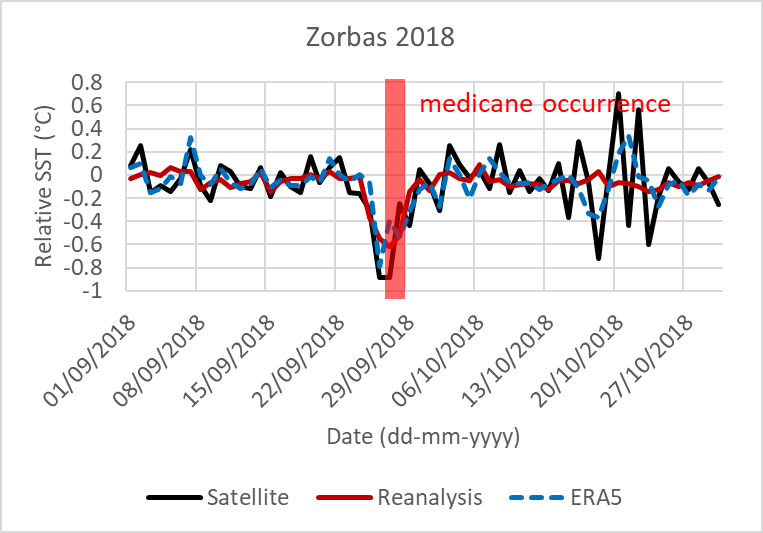  (b) |
| --- | --- |

Fig. S16 Analysis on the SST for the Medicane Zorbas (27-30 September 2018) and relative occurrence reported in red bar; (a) SST time-series; (b) relative SST time-series.

| 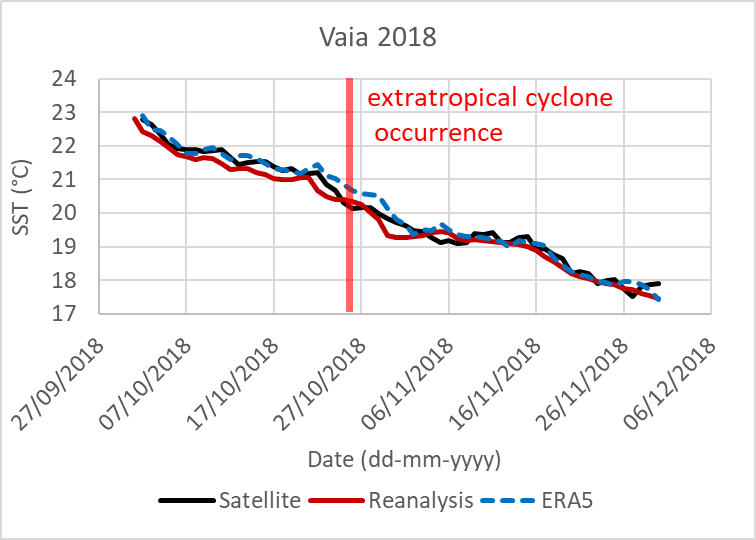  (a) | 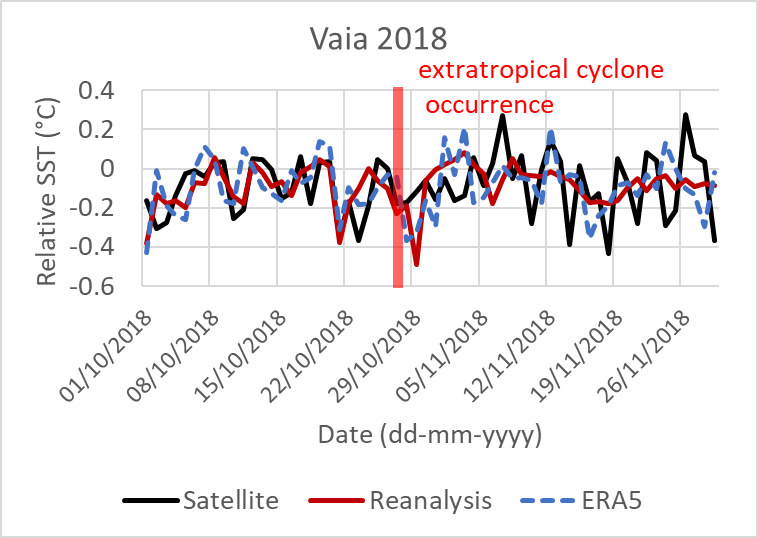  (b) |
| --- | --- |

Fig. S17 Analysis on the SST for the extratropical cyclone Vaia (28-29 October 2018) and relative occurrence reported in red bar; (a) SST time-series; (b) relative SST time-series.

| 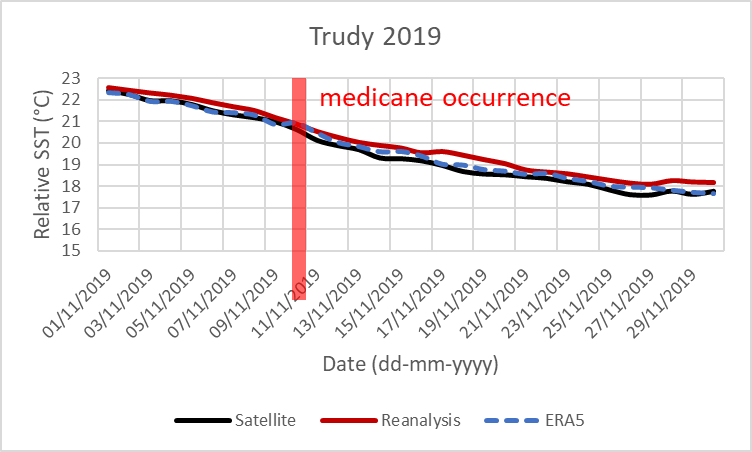  (a) | 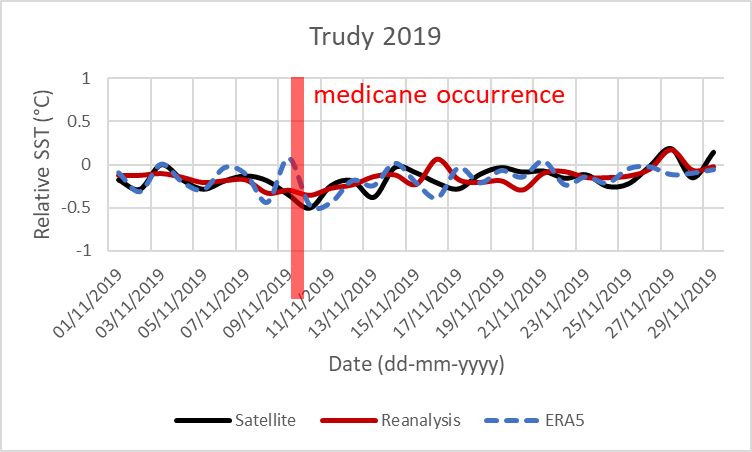  (b) |
| --- | --- |

Fig. S18 Analysis on the SST for the Medicane Trudy (11-13 November 2019) and relative occurrence reported in red bar; (a) SST time-series; (b) relative SST time-series.

| 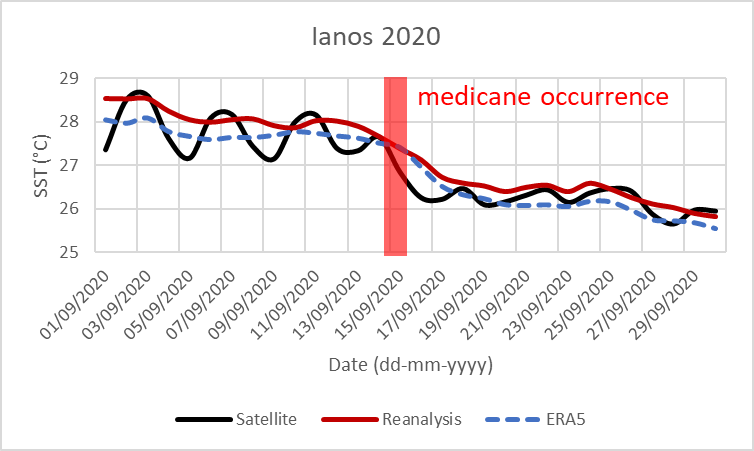  (a) | 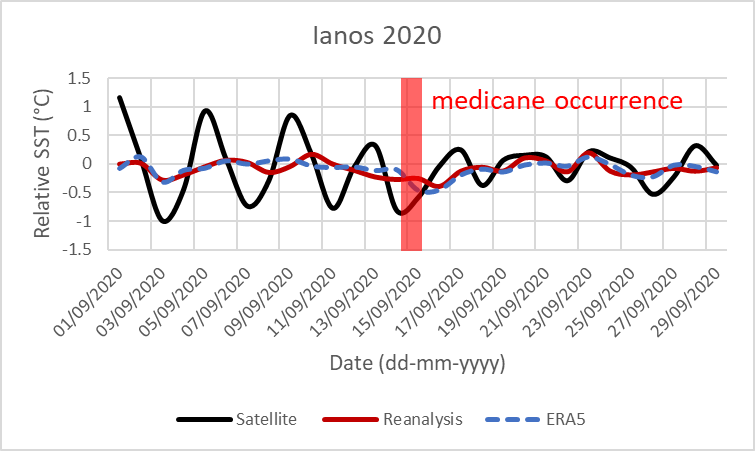  (b) |
| --- | --- |

Fig. S19 Analysis on the SST for the Medicane Ianos (15-20 September 2020) and relative occurrence reported in red bar; (a) SST time-series; (b) relative SST time-series.

| 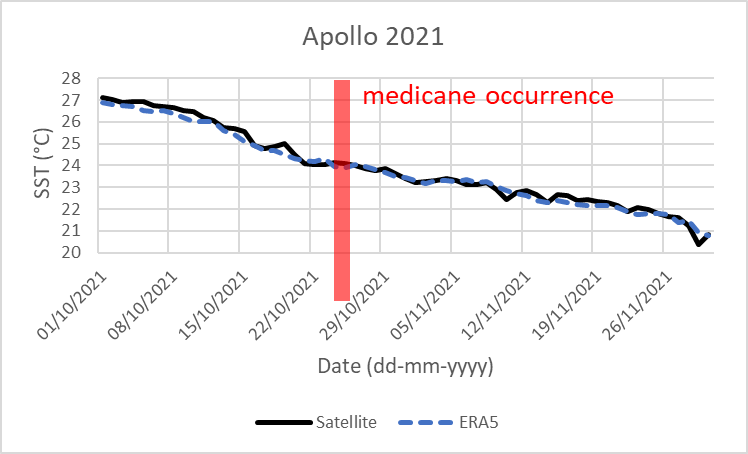  (a) | 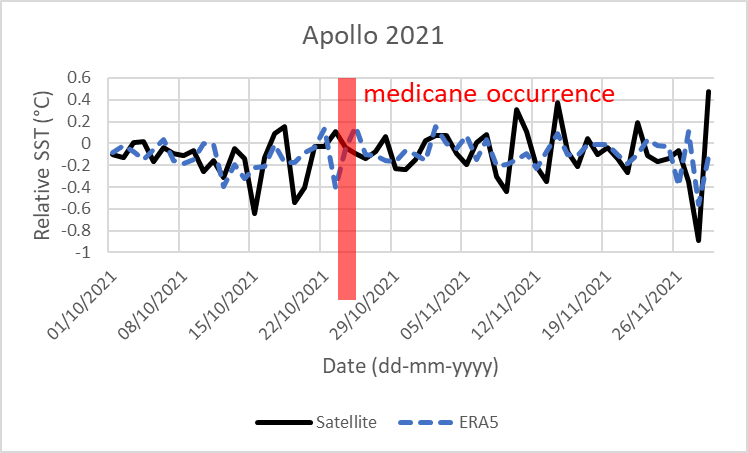  (b) |
| --- | --- |

Fig. S20 Analysis on the SST for the Medicane Apollo (25 October - 01 November 2021) and relative occurrence reported in red bar; (a) SST time-series; (b) relative SST time-series.

| 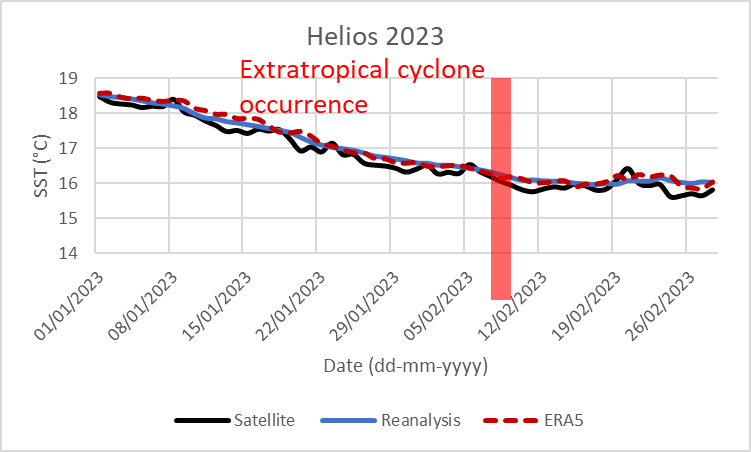  (a) | 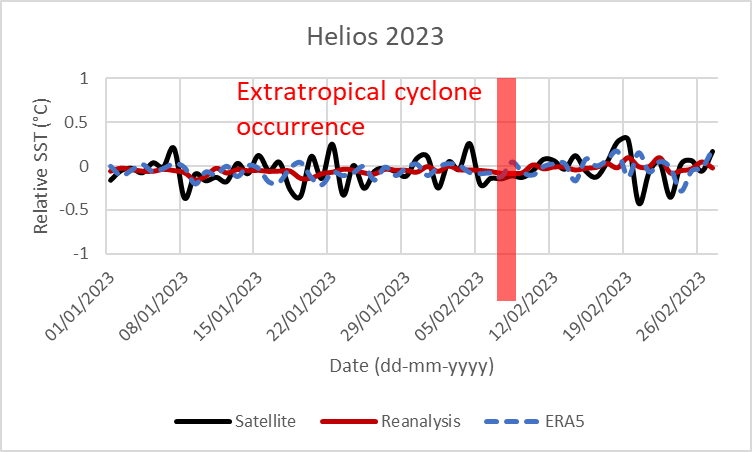  (b) |
| --- | --- |

Fig. S21 Analysis on the SST for the Medicane Helios (08 - 11 February 2023) and relative occurrence reported in red bar; (a) SST time-series; (b) relative SST time-series.

| 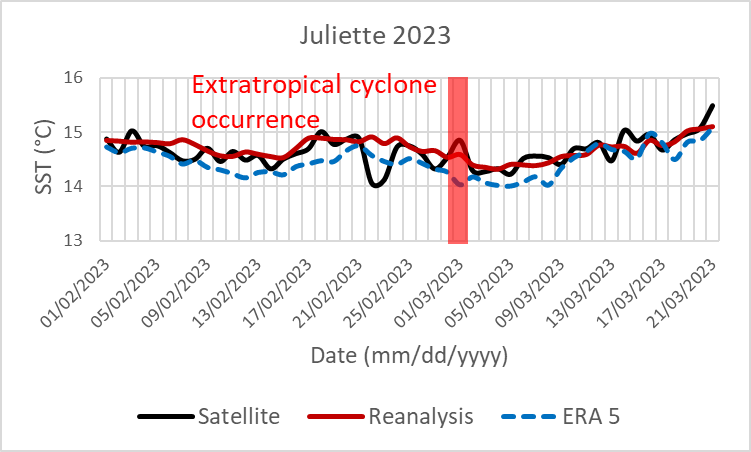  (a) | 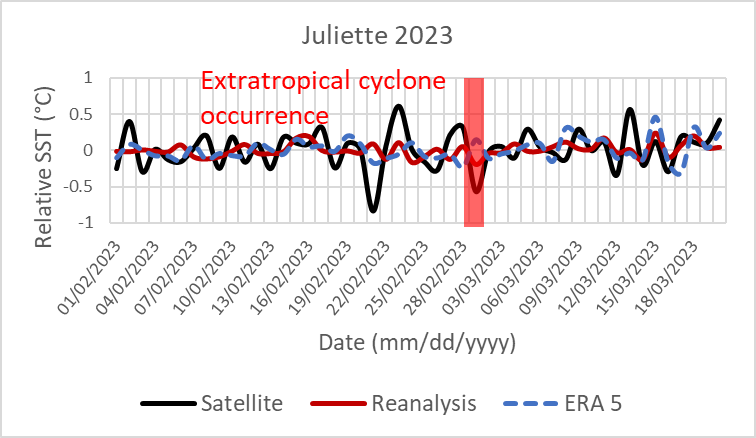  (b) |
| --- | --- |

Fig. S22 Analysis on the SST for the Medicane Juliette (28 February - 02 March 2023) and relative occurrence reported in red bar; (a) SST time-series; (b) relative SST time-series.

| 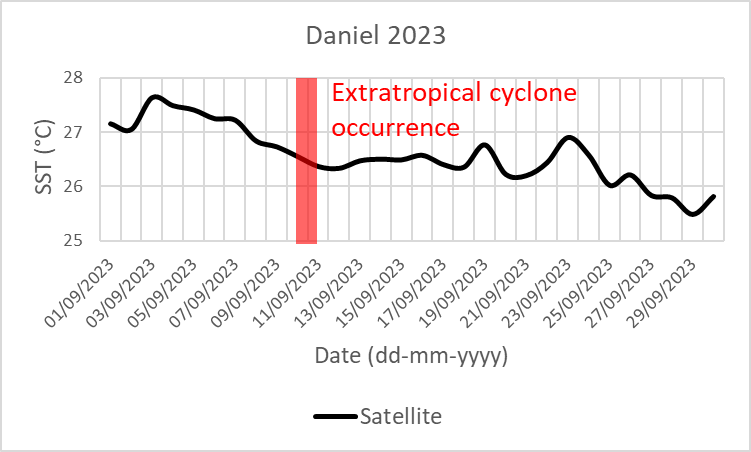  (a) | 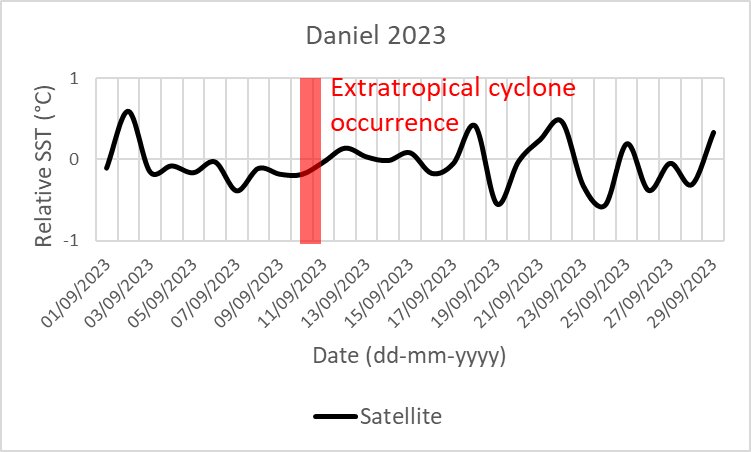  (b) |
| --- | --- |

Fig. S23 Analysis on the SST for the Medicane Daniel (10-12 September 2023) and relative occurrence reported in red bar; (a) SST time-series; (b) relative SST time-series.

**1.3 Mean composite maps of the main Mediterranean cyclones**

Here, mean composite maps of the main Mediterranean cyclones are reported in the following manner:

- Fig.n a) – SST map 10 day prior to the cyclone occurrence;
- Fig.n b) – SST map at the time of the cyclone occurrence;
- Fig.n c) – map of the thermal changes.

**Medicane Celeno (1995)**

**
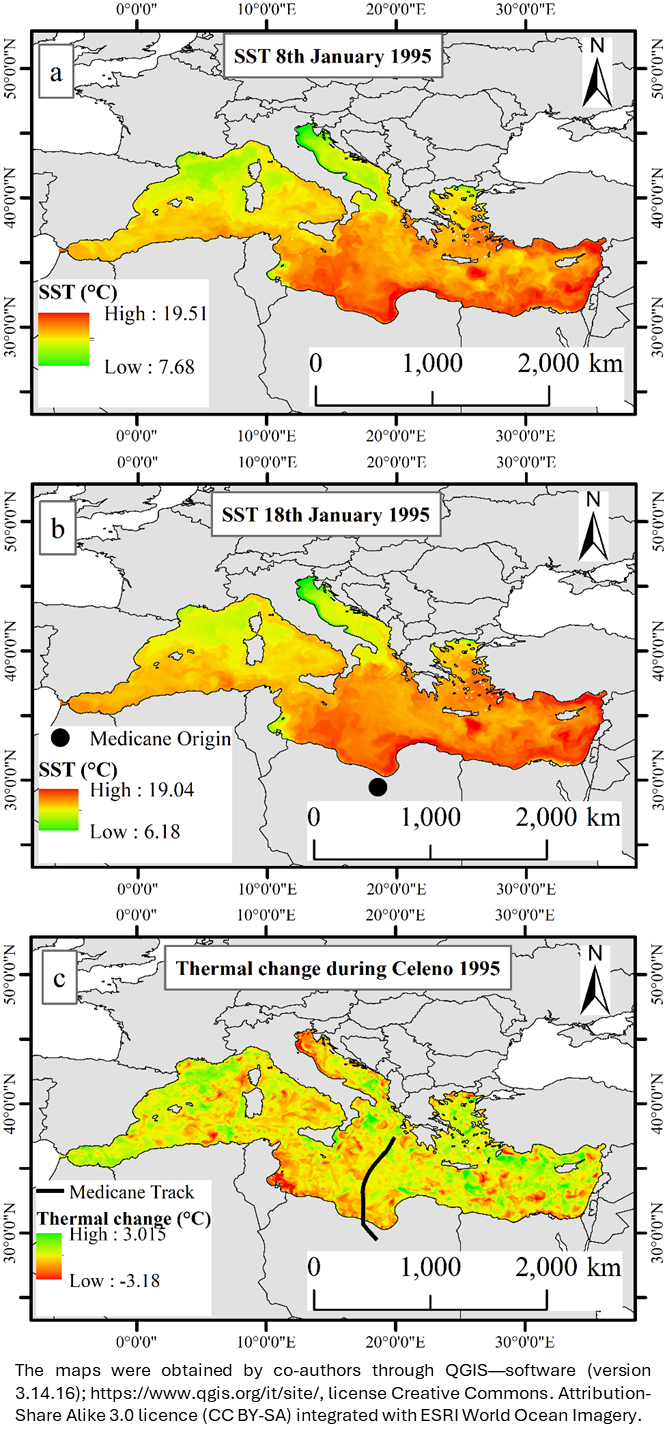
**

Fig. S24 Celeno (1995), a) SST 10 days before onset b) SST during onset c) Thermal difference of 10days SST.

**Medicane Cornelia (1996)**

**
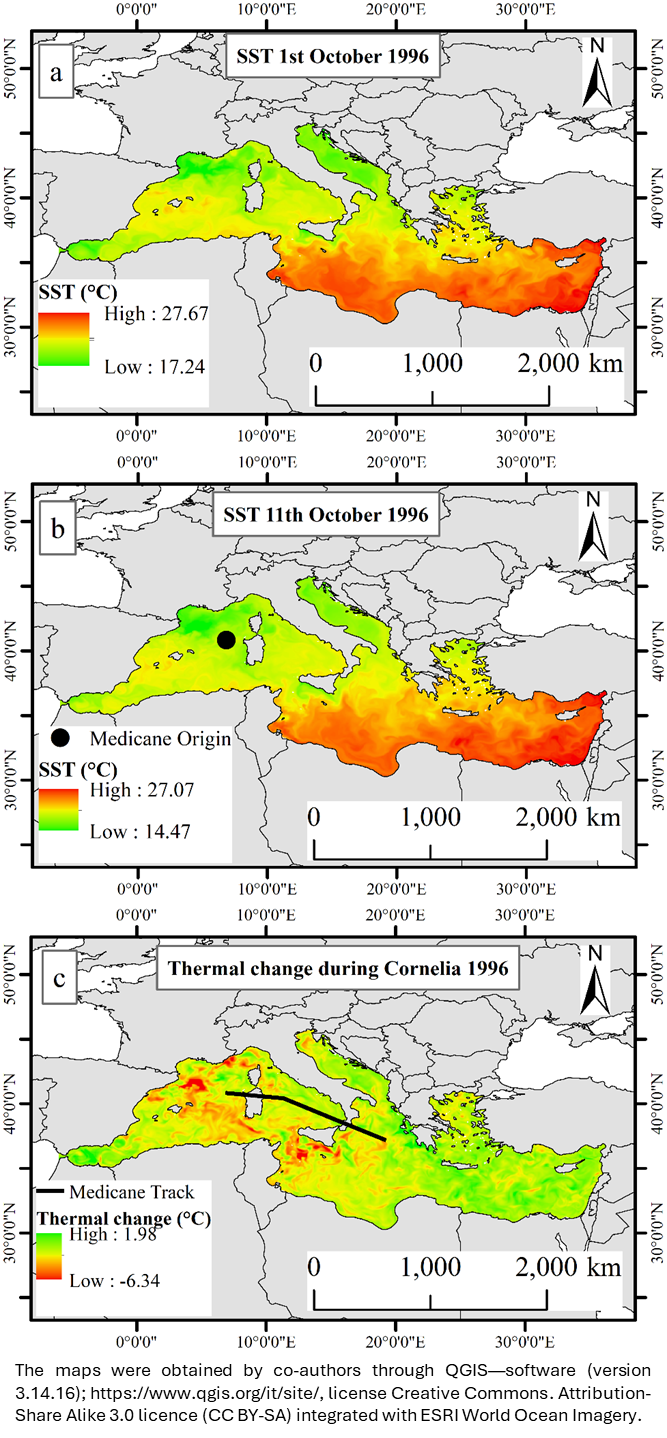
**

Fig. S25 Cornelia (1996), a) SST 10 days before onset b) SST during onset c) Thermal difference of 10days SST.

**Medicane Zeo (2005)**

**
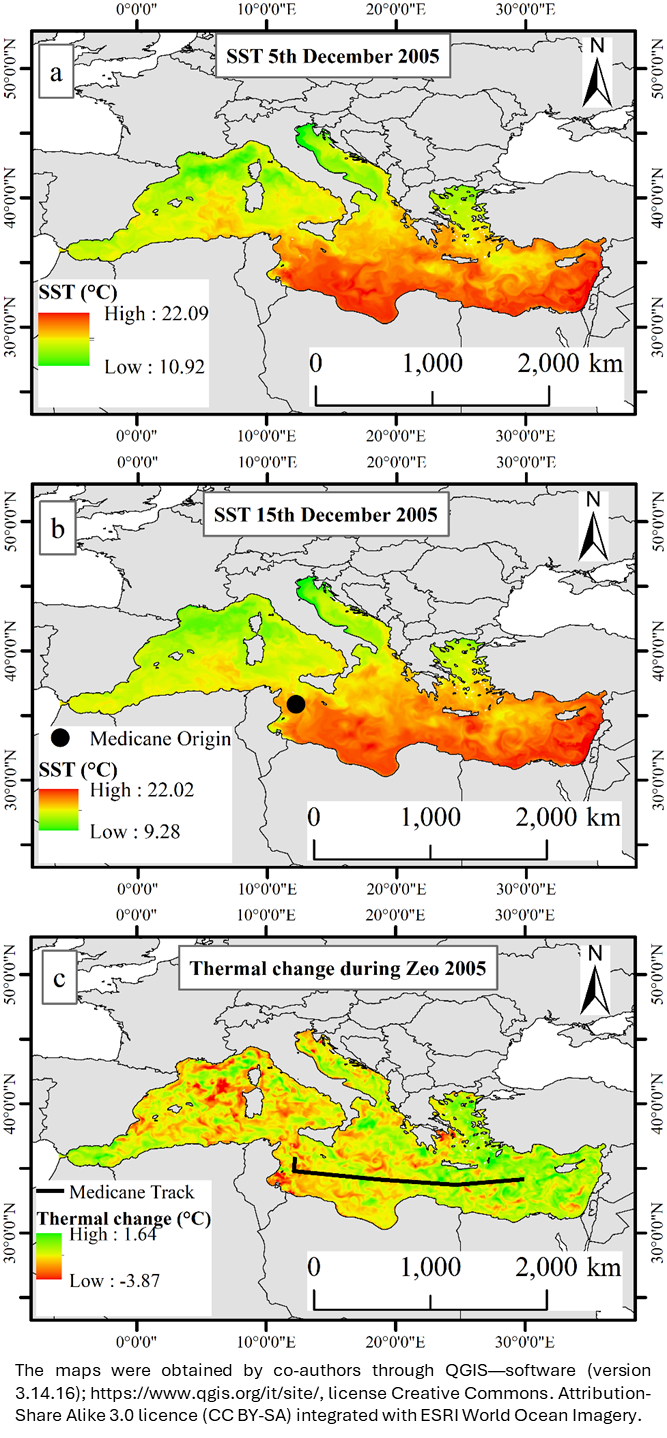
**

Fig. S26 Zeo (2005), a) SST 10 days before onset b) SST during onset c) Thermal difference of 10days SST.

**Medicane Rolph (2011)**

**
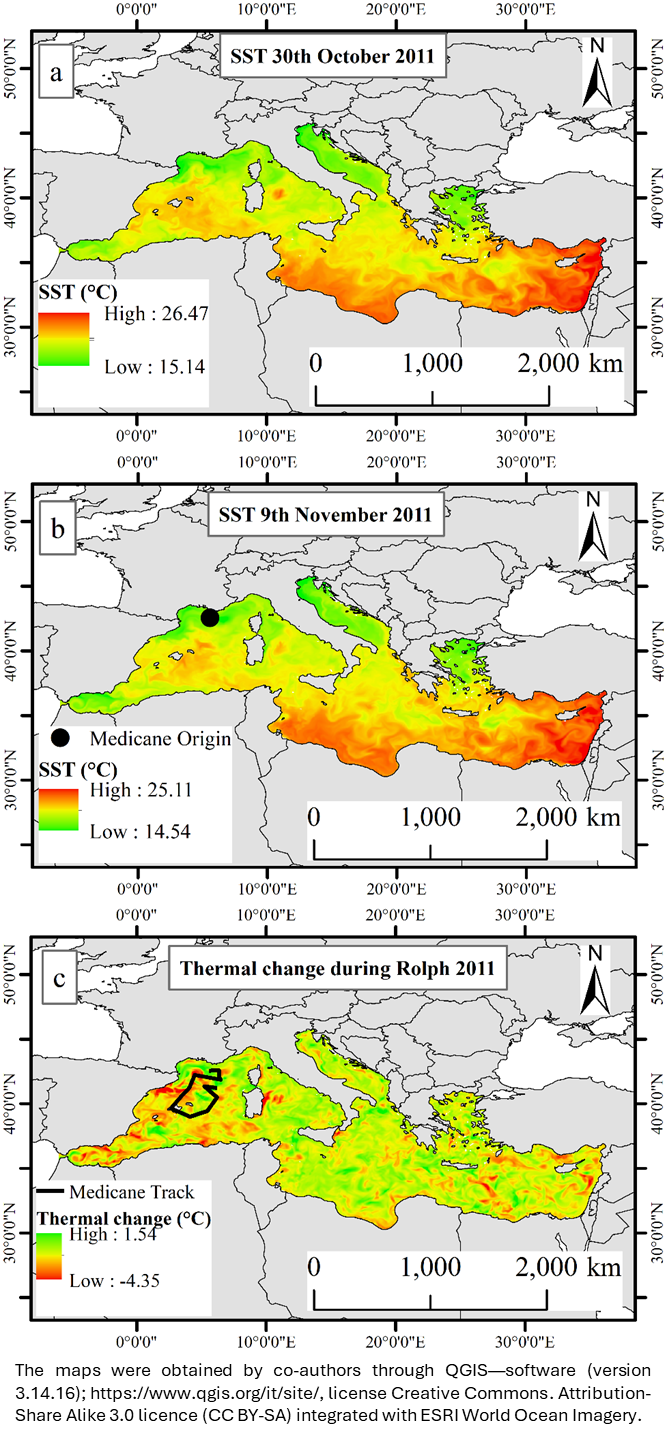
**

Fig. S27 Rolph (2011), a) SST 10 days before onset b) SST during onset c) Thermal difference of 10days SST.

**Medicane Qendresa (2014)**

**
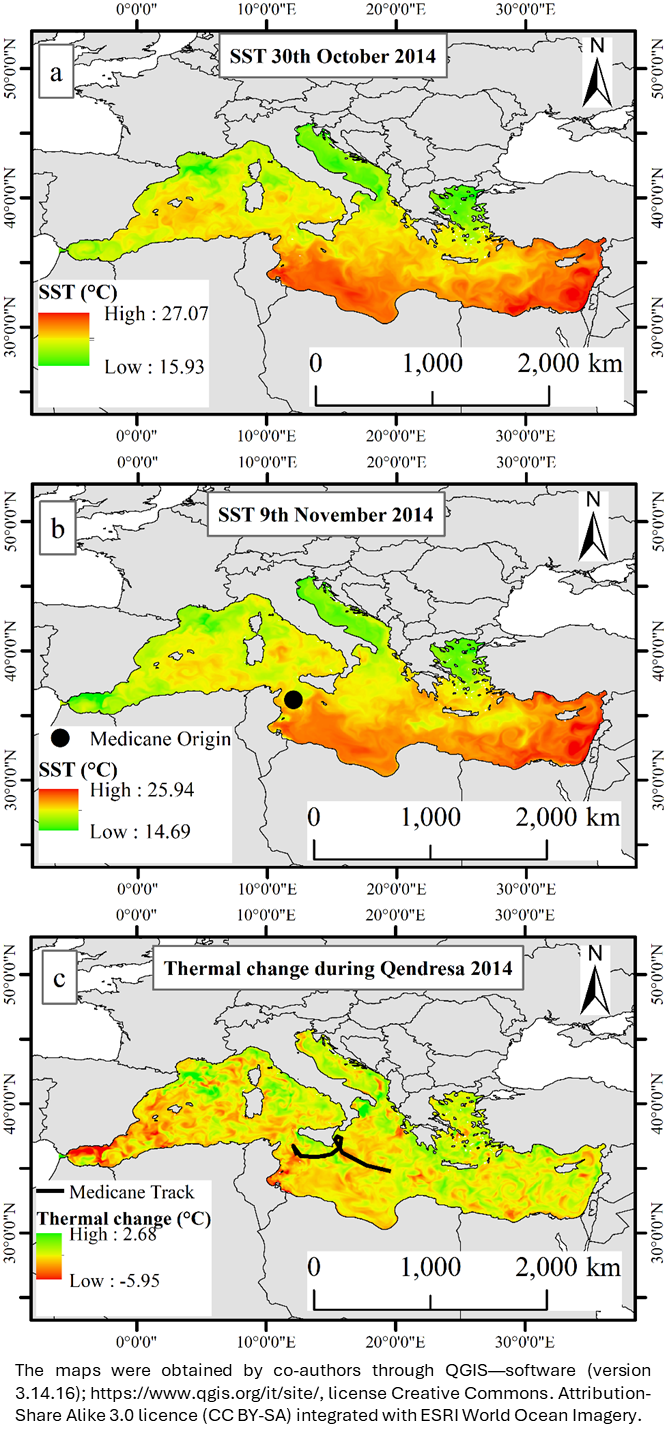
**

Fig.S28 Qendresa (2014), a) SST 10 days before onset b) SST during onset c) Thermal difference of 10days SST.

**Medicane Zorbas (2018)**

**
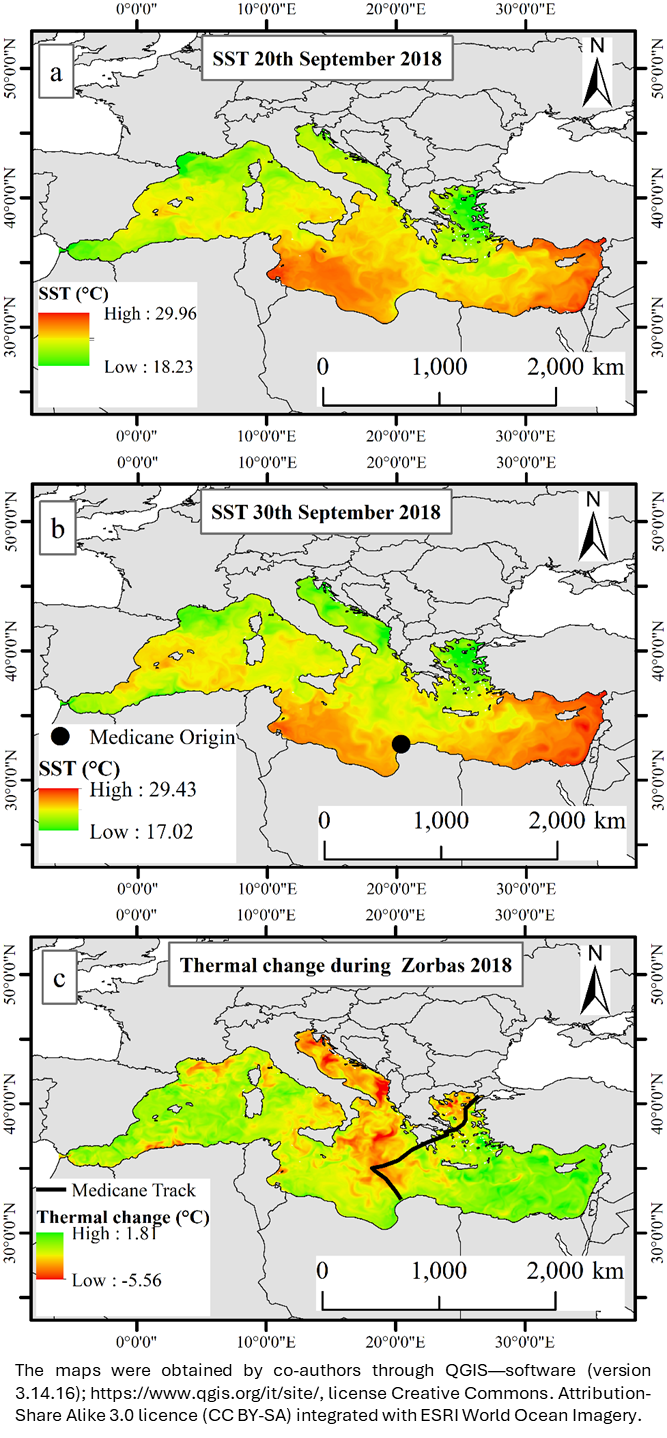
**

Fig.S29 Zorbas (2018), a) SST 10 days before onset b) SST during onset c) Thermal difference of 10days SST.

**Cyclone Vaia (2018)**

**
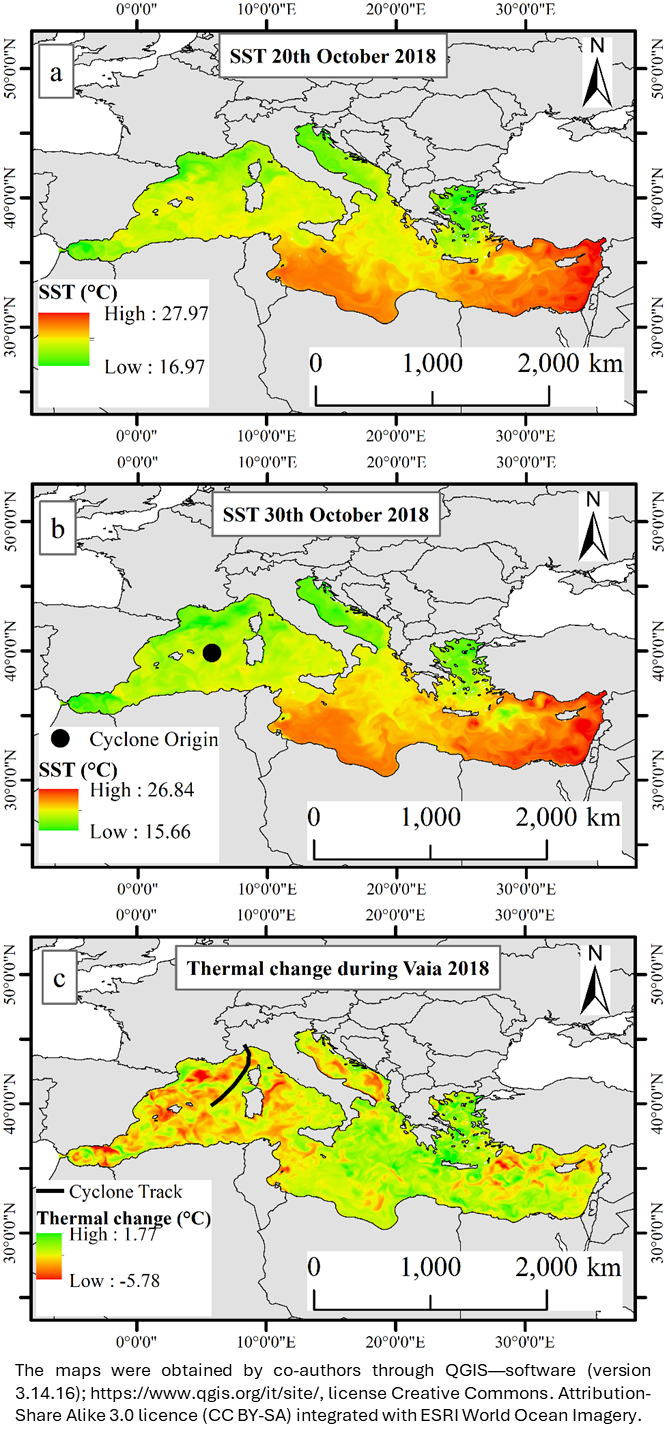
**

Fig.S30 Vaia (2018), a) SST 10 days before onset b) SST during onset c) Thermal difference of 10days SST.

**Medicane Trudy (2019)**

**
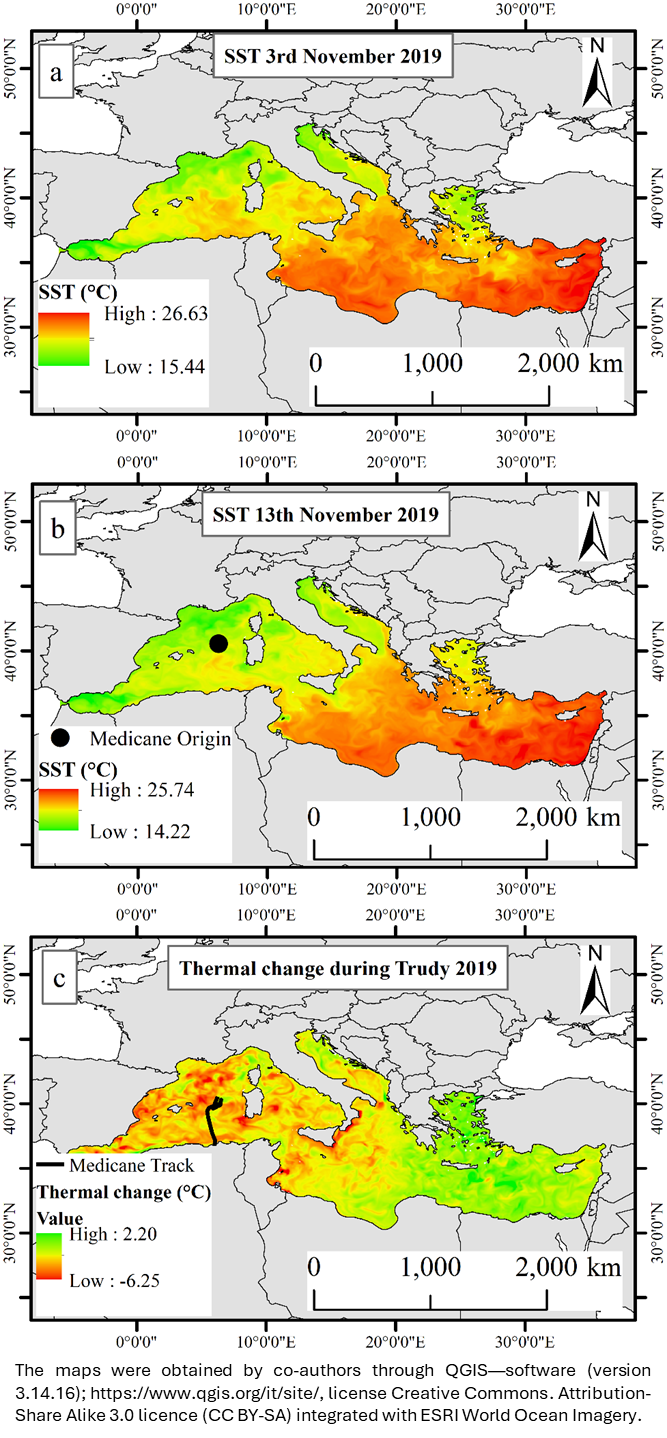
**

Fig. S31 Trudy (2019), a) SST 10 days before onset b) SST during onset c) Thermal difference of 10days SST.

**Medicane Ianos (2020)**

**
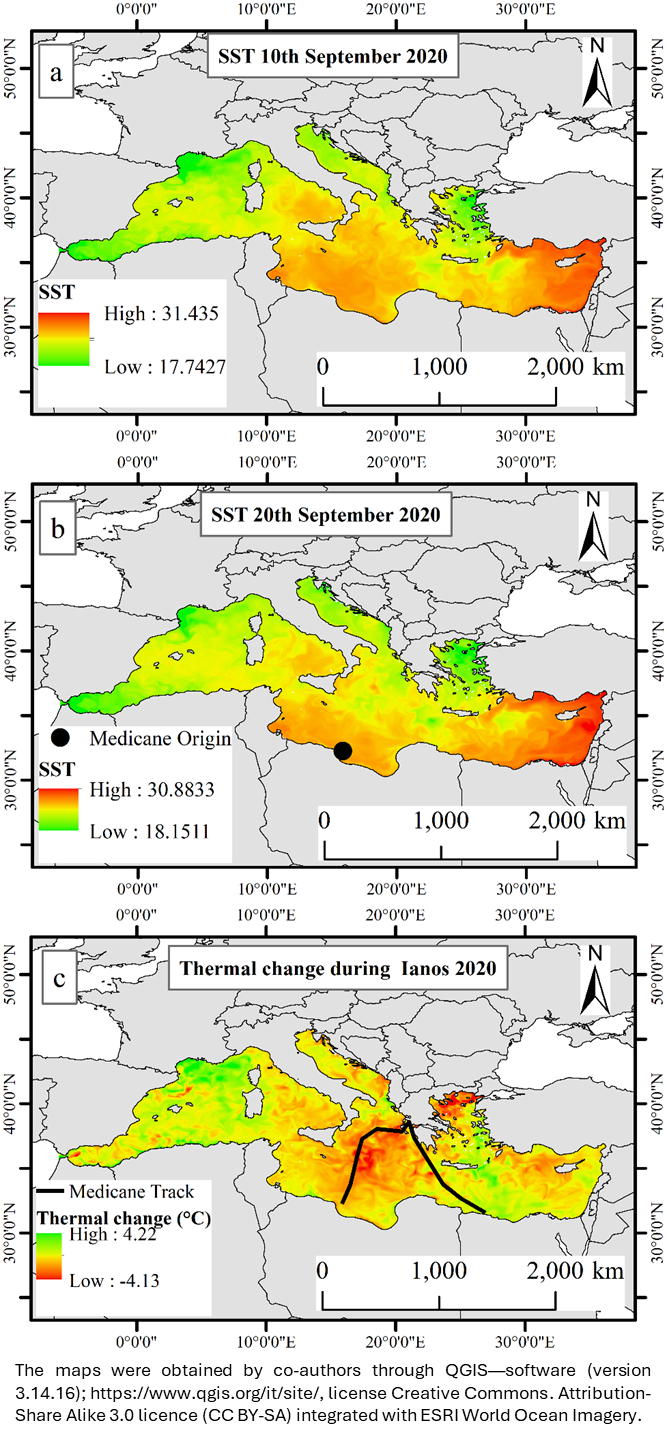
**

Fig. S32 Ianos (2020), a) SST 10 days before onset b) SST during onset c) Thermal difference of 10days SST.

**Medicane Apollo (2021)**

**
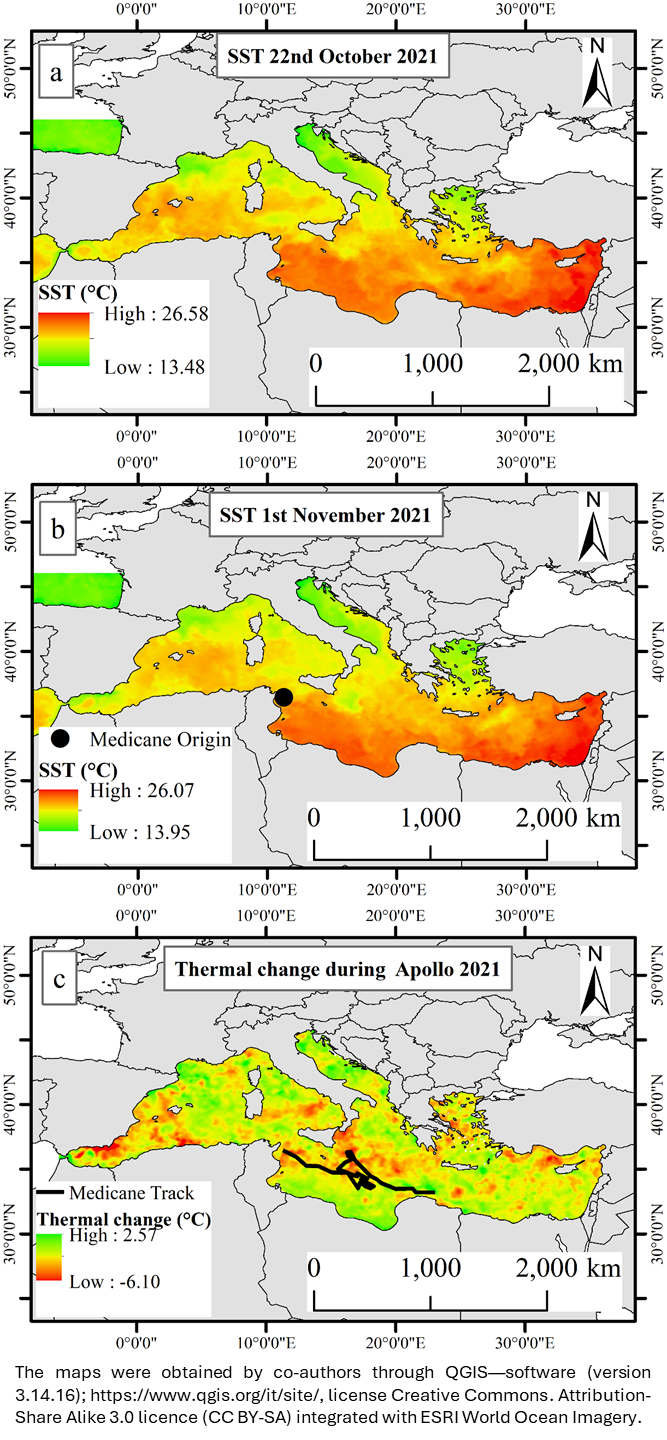
**

Fig. S33 Apollo (2021), a) SST 10 days before onset b) SST during onset c) Thermal difference of 10days SST.

**Cyclone Blas (2021)**

**
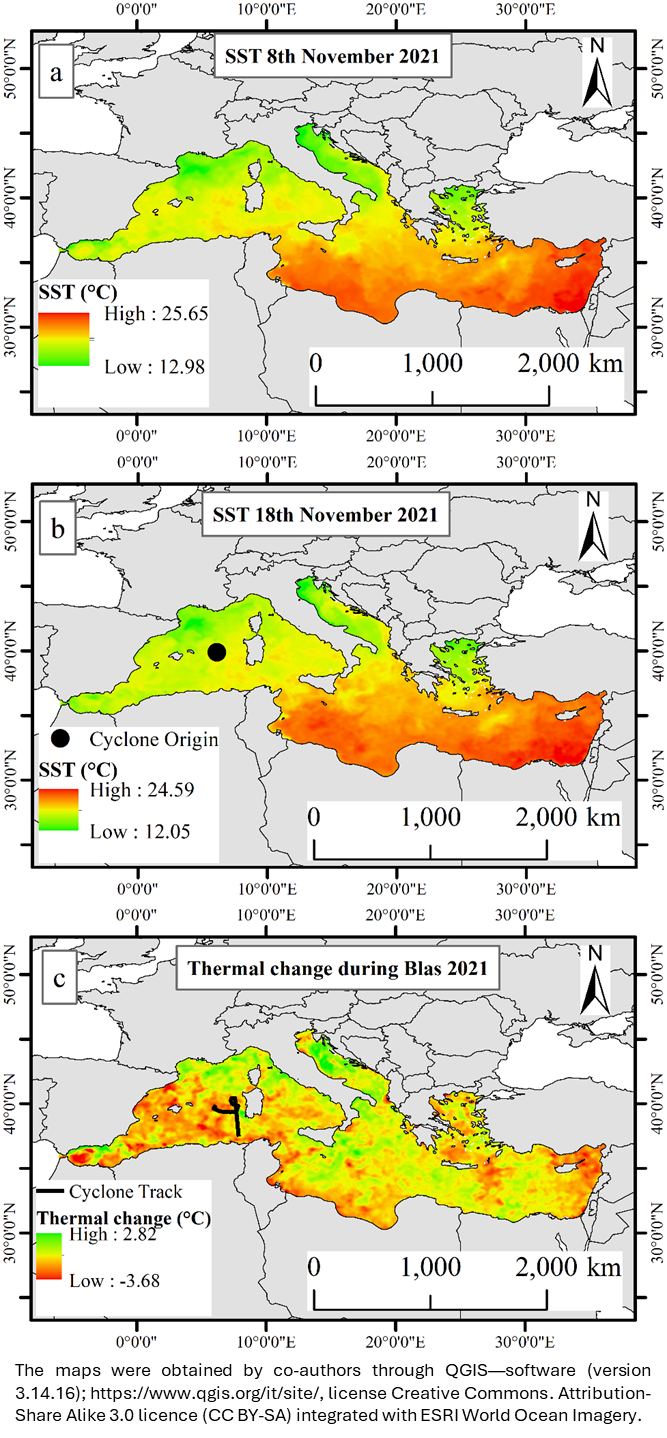
**

Fig. S34 Blas (2021), a) SST 10 days before onset b) SST during onset c) Thermal difference of 10days SST.

**Cyclone Helios (2023)**

**
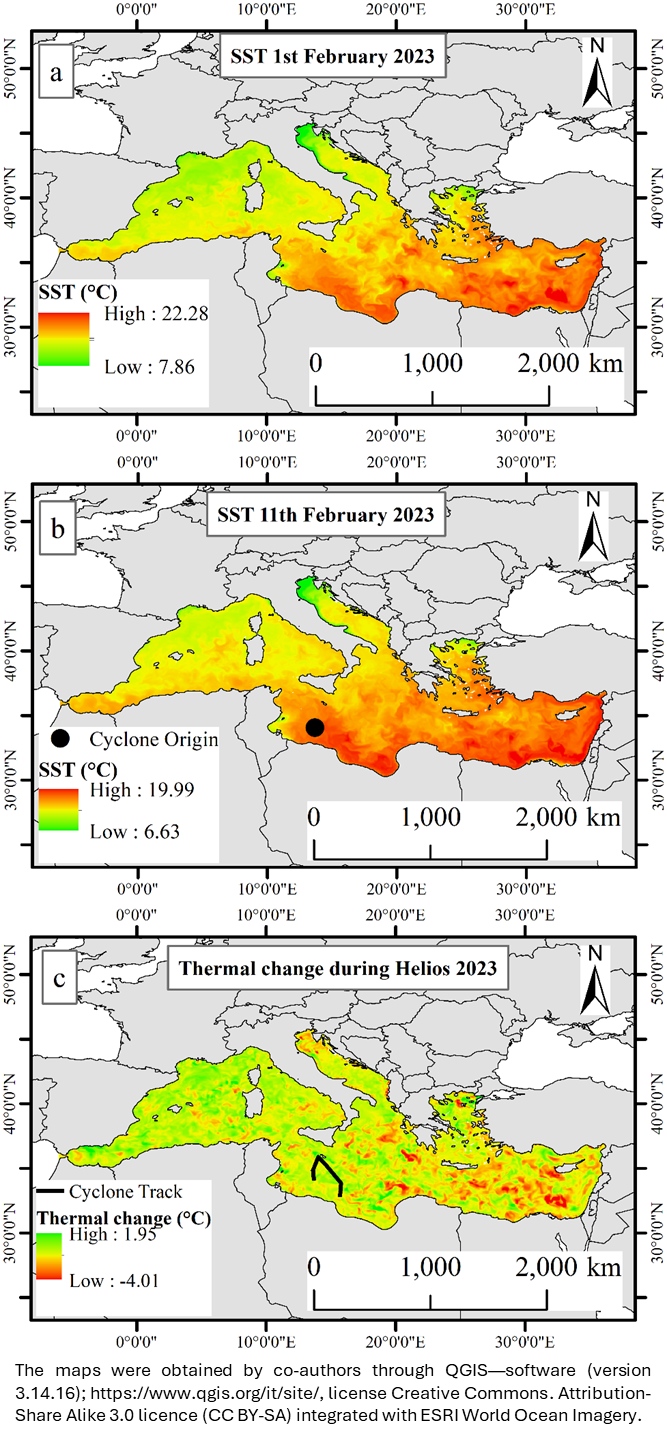
**

Fig.S35 Helios (2023), a) SST 10 days before onset b) SST during onset c) Thermal difference of 10days SST.

**Cyclone Juliette (2023)**

**
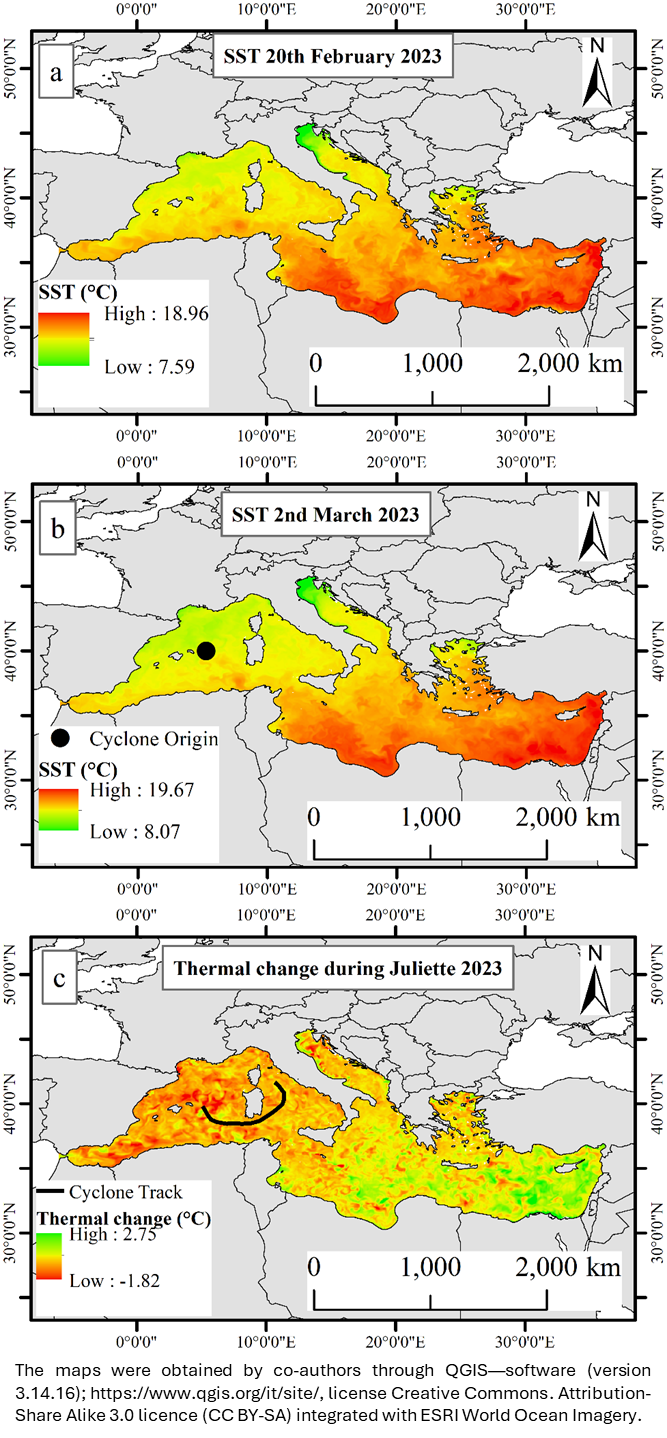
**

Fig. S36 Juliette (2023), a) SST 10 days before onset b) SST during onset c) Thermal difference of 10days SST.

**Cyclone Daniel (2023)**

**
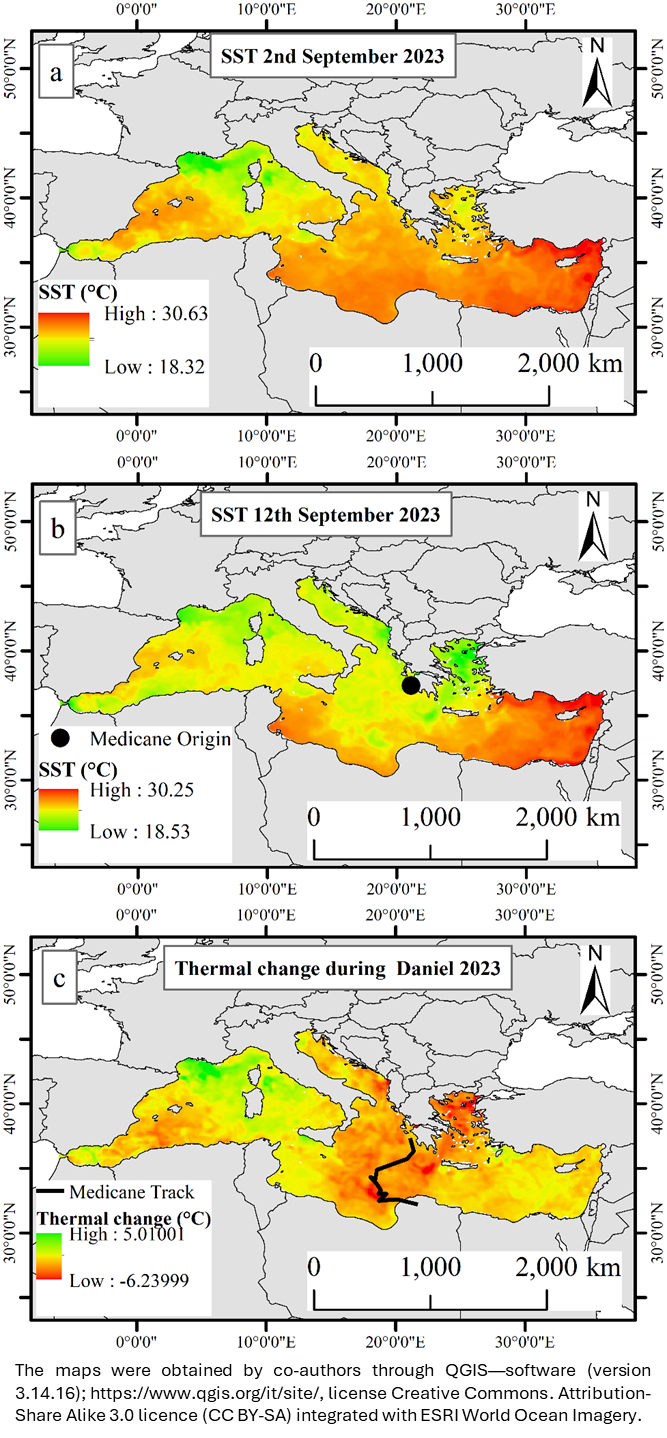
**

Fig. S37 Daniel (2023), a) SST 10 days before onset b) SST during onset c) Thermal difference of 10days SST.

Reference

1. Reale, O. Dynamics and classification of two sub-syn-optic scale ‘Hurricane-like’ vortices over theMediterranean Sea. *In Annales Geophysicae. Part II:Hydrology, Oceans & Atmosphere* **(Supplement II to Volume 16), EGS**, C634 (1998).

2. Flaounas, E. *et al.* A composite approach to produce reference datasets for extratropical cyclone tracks: application to Mediterranean cyclones. *Weather and Climate Dynamics* **4**, 639–661 (2023).

3. Picornell, M. A., Campins, J. & Jansà, A. Detection and thermal description of medicanes from numerical simulation. *Natural Hazards and Earth System Sciences* **14**, 1059–1070 (2014).

4. Tous, M. & Romero, R. Meteorological environments associated with medicane development. *International Journal of Climatology* **33**, 1–14 (2013).

5. Cavicchia, L. & von Storch, H. The simulation of medicanes in a high-resolution regional climate model. *Clim Dyn* **39**, 2273–2290 (2012).

6. Lagouvardos, K., Kotroni, V., S Nickovic, D. J., Kallos, G. & Tremback, C. J. Observations and model simulations of a winter sub-synoptic vortex over the central Mediterranean. *Meteorological Applications* **6**, 371–383 (1999).

7. Emanuel, K. Genesis and maintenance of ‘Mediterranean hurricanes’. in *Advances in Geosciences* vol. 2 217–220 (Copernicus GmbH, 2005).

8. Mazza, E., Ulbrich, U. & Klein, R. The Tropical Transition of the October 1996 Medicane in the Western Mediterranean Sea: A Warm Seclusion Event. *Monthly Weather Review* **145**, 2575–2595 (2017).

9. Fita, L. & Flaounas, E. Medicanes as subtropical cyclones: the December 2005 case from the perspective of surface pressure tendency diagnostics and atmospheric water budget. *Quarterly Journal of the Royal Meteorological Society* **144**, 1028–1044 (2018).

10. Lira-Loarca, A., Cáceres-Euse, A., De-Leo, F. & Besio, G. Wave modeling with unstructured mesh for hindcast, forecast and wave hazard applications in the Mediterranean Sea. *Applied Ocean Research* **122**, 103118 (2022).

11. Pravia-Sarabia, E., Gómez-Navarro, J. J., Jiménez-Guerrero, P. & Montávez, J. P. Influence of sea salt aerosols on the development of Mediterranean tropical-like cyclones. *Atmospheric Chemistry and Physics* **21**, 13353–13368 (2021).

12. Tropical storm develops in the Mediterranean Sea | EUMETSAT. https://www.eumetsat.int/tropical-storm-develops-mediterranean-sea.

13. Di Muzio, E., Riemer, M., Fink, A. H. & Maier-Gerber, M. Assessing the predictability of Medicanes in ECMWF ensemble forecasts using an object-based approach. *Quarterly Journal of the Royal Meteorological Society* **145**, 1202–1217 (2019).

14. Bouin, M.-N. & Lebeaupin Brossier, C. Surface processes in the 7 November 2014 medicane from air–sea coupled high-resolution numerical modelling. *Atmospheric Chemistry and Physics* **20**, 6861–6881 (2020).

15. Jangir, B., Mishra, A. K. & Strobach, E. Effects of Mesoscale Eddies on the Intensity of Cyclones in the Mediterranean Sea. *Journal of Geophysical Research: Atmospheres* **128**, e2023JD038607 (2023).

16. Di Muzio, E., Riemer, M., Fink, A. H. & Maier-Gerber, M. Assessing the predictability of Medicanes in ECMWF ensemble forecasts using an object-based approach. *Quarterly Journal of the Royal Meteorological Society* **145**, 1202–1217 (2019).

17. Lagouvardos, K., Karagiannidis, A., Dafis, S., Kalimeris, A. & Kotroni, V. Ianos—A Hurricane in the Mediterranean. *Bulletin of the American Meteorological Society* **103**, E1621–E1636 (2022).

18. Marra, A. C. *et al.* The Precipitation Structure of the Mediterranean Tropical-Like Cyclone Numa: Analysis of GPM Observations and Numerical Weather Prediction Model Simulations. *Remote Sensing* **11**, 1690 (2019).

19. Scicchitano, G. *et al.* Comparing impact effects of common storms and Medicanes along the coast of south-eastern Sicily. *Marine Geology* **439**, 106556 (2021).

20. Portmann, R., González-Alemán, J. J., Sprenger, M. & Wernli, H. Medicane Zorbas: Origin and impact of an uncertain potential vorticity streamer. *Weather and Climate Dynamics Discussions* 1–30 (2019) doi:https://doi.org/10.5194/wcd-2019-1.

21. Scicchitano, G. *et al.* The First Video Witness of Coastal Boulder Displacements Recorded during the Impact of Medicane “Zorbas” on Southeastern Sicily. *Water* **12**, 1497 (2020).

22. Kouroutzoglou, J. *et al.* Analysis of the Transition of an Explosive Cyclone to a Mediterranean Tropical-like Cyclone. *Atmosphere* **12**, 1438 (2021).

23. Davolio, S., Fera, S. D., Laviola, S., Miglietta, M. M. & Levizzani, V. Heavy Precipitation over Italy from the Mediterranean Storm “Vaia” in October 2018: Assessing the Role of an Atmospheric River. *Monthly Weather Review* **148**, 3571–3588 (2020).

24. Cavaleri, L., Barbariol, F., Bertotti, L., Besio, G. & Ferrari, F. The 29 October 2018 storm in Northern Italy: Its multiple actions in the Ligurian Sea. *Progress in Oceanography* **201**, 102715 (2022).

25. Zekkos, D. *et al.* *The September 18-20 2020 Medicane Ianos Impact on Greece Phase I Reconnaissance Report*. (2020). doi:10.18118/G6MT1T.

26. Borzì, A. M. *et al.* Monitoring extreme meteo-marine events in the Mediterranean area using the microseism (Medicane Apollo case study). *Sci Rep* **12**, 21363 (2022).

27. Faranda, D. *et al.* A climate-change attribution retrospective of some impactful weather extremes of 2021. *Weather and Climate Dynamics* **3**, 1311–1340 (2022).

28. Borzì, A. *et al.* *Integration of microseism, wavemeter buoy, HF Radar and hindcast data to analyze the Mediterranean cyclone Helios*. (2023). doi:10.5194/egusphere-2023-1212.

29. D’Adderio, L. P. *et al.* Helios and Juliette: Two Falsely Acclaimed Medicanes. SSRN Scholarly Paper at https://doi.org/10.2139/ssrn.4542818 (2023).
